# Listerin Alleviates Alzheimer's Disease through IRE1‐mediated Decay of *TLR4* mRNA

**DOI:** 10.1002/advs.202414956

**Published:** 2025-05-31

**Authors:** Fei Qin, Runyu Cao, Xuemei Bai, Jiahua Yuan, Wanwei Sun, Yi Zheng, Xiaopeng Qi, Wei Zhao, Bingyu Liu, Chengjiang Gao

**Affiliations:** ^1^ Key Laboratory of Infection Immunity and prevention of Shandong Province & Key Laboratory for Experimental Teratology of Ministry of Education Shandong University Jinan Shandong 250012 P. R. China; ^2^ Department of Immunology School of Basic Medical Sciences Shandong University Jinan Shandong 250012 P. R. China; ^3^ Advanced Medical Research Institute Cheeloo College of Medicine Shandong University Jinan Shandong 250012 P. R. China; ^4^ Department of Pathogenic Biology School of Basic Medical Sciences Shandong University Jinan Shandong 250012 P. R. China

**Keywords:** Alzheimer's disease, inflammation, IRE1α‐dependent decay (RIDD), Listerin, TLR4

## Abstract

Alzheimer's disease (AD) is the most prevalent neurodegenerative disorder, accounting for ≈60–70% of all dementia cases worldwide. Microglial‐mediated brain inflammation is thought to play key roles in AD progression. Clinical evidence and animal models have indicated that the ribosome‐associated quality control (RQC) component Listerin is involved in the development of AD. How Listerin regulates the development and progression of AD is unknown. Here, it is demonstrated that Listerin can decrease brain inflammation and alleviate AD‐related cognitive impairments. Microglial‐specific knockout of Listerin exhibits deteriorative cognitive symptoms based on the extracellular Amyloid‐β (Aβ) or Lipopolysaccharide (LPS) injection. Mechanistically, Listerin directly binds to Toll‐like receptor 4 (TLR4) mRNA and facilitates the IRE1α‐mediated cleavage and degradation of *TLR4* mRNA, leading to the alleviation of TLR4‐induced brain inflammation. Adenovirus‐mediated overexpression of Listerin decelerates the disease progression in the mouse model of Aβ‐mediated neurodegeneration. Thus, Listerin is an important suppressor of microglia‐induced brain inflammation and may be a potential therapeutic target for AD treatment.

## Graphical abstract

Alzheimer's disease (AD) progression is influenced by microglia‐mediated neuroinflammation. Here, it is demonstrated that Listerin suppresses neuroinflammatory signaling and cognitive impairment in AD models by triggering IRE1α‐mediated TLR4 mRNA decay. Adenoviral Listerin delivery reduces amyloid‐β pathology, positioning it as a new therapeutic target.

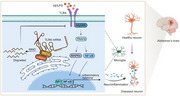

## 1 Introduction

Alzheimer's disease (AD) is a neurodegenerative disorder characterized by early progressive memory loss followed by impaired executive function and other behavioral disorders, including agitation and paranoia.^[^ ^1^ ^]^ The pathological hallmarks of the brains in Alzheimer's disease patients are the accumulation of beta‐amyloid plaques consisting of beta‐amyloid (Aβ) outside neurons and abnormal form of neurofibrillary tangles made up of misfolded, hyperphosphorylated Tau protein inside neurons.^[^ ^2^ ^]^ AD is generally prevalent among older adults worldwide, however, the physiological and pathological molecular signaling mechanisms of AD are yet to be fully understood. This has impeded the development of effective therapeutic strategies.

Currently, accumulating studies have shown that brain inflammation has a great impact on the occurrence and development of AD, which revealed the potential chances for research of new possible therapeutic options.^[^ ^3^ ^]^ Microglia, the resident immune cells of the central nervous system, play a crucial role in maintaining neural homeostasis through dynamical dual regulation mechanisms of neuroinflammation. Under physiological conditions, microglia constantly survey the brain microenvironment and respond to various stimuli.^[^ ^4^ ^]^ However, under pathological conditions, microglia activate via pattern recognition receptors (TLRs, NLRs), releasing pro‐inflammatory cytokines (TNF‐α, IL‐1β, IL‐6) to initiate immune responses and recruit peripheral immune cells, often exacerbating neurodegeneration in diseases like Alzheimers.^[^ ^5^ ^]^ This sustained inflammatory response contributes to neuronal damage, synaptic dysfunction, and disease progression. For example, abnormal A β can stimulate TLR4 receptors to activate microglia and increase the expression of inflammatory factors, leading to abnormal brain inflammation, which induces microtubule‐associated protein Tau hyperphosphorylation and cell apoptosis in neurons, resulting in specific functional impairment phenotypes.^[^ ^6^ ^]^ Moreover, persistent chronic inflammatory activity in the brain could create a positive feedback loop in AD pathogenesis by accelerating Aβ production and inducing Tau phosphorylation and diffusion.^[^ ^3^, ^7^ ^]^ Until now, the signaling loops through which brain inflammation interacts with AD pathology need to be further explored.

The unfolded protein response (UPR), which mediates the endoplasmic reticulum (ER) proteostasis surveillance, is a comprehensive signal transduction pathway that maintains the balance of protein in the ER during physiological or pathophysiological unfolded protein stress.^[^ ^8^ ^]^ The activation of the UPR is mainly triggered by three ER transmembrane receptors, PERK, IRE1α, and ATF6, in which IRE1α has been reported to be a primary regulator of cell fate determination during ER stress.^[^ ^9^ ^]^ In earlier studies of the UPR, IRE1α induced the transcription of ER quality control components by splicing the mRNA of X‐box binding protein‐1 (XBP‐1), restoring ER homeostasis and promoting cell survival.^[^ ^10^ ^]^ Some interesting studies have revealed that several more extensive mRNAs have been identified as human IRE1α splicing substrates. IRE1α‐dependent decay (RIDD) mainly targets ER‐bound mRNAs, and seems to require a typical stem‐loop endo‐motif, the XBP1u‐like consensus loop sequence CNG|CAGN, supported by stable stem.^[^ ^11^ ^]^ Enhanced IRE1α signaling has been observed in AD patient neurons containing abnormally phosphorylated Tau.^[^ ^12^ ^]^ However, the underlying mechanism of IRE1 α‐ dependent regulates the occurrence and development of AD is still unclear, and further in‐depth exploration is needed.

The E3 ubiquitin ligase Listerin (LTN1 in yeast), a pivotal component in the ribosome‐associated protein quality control (RQC) system, predominantly marks and degrades abnormally synthesized proteins through the proteasome pathway.^[^ ^13^ ^]^ Previous studies on RQC have been conducted mainly in yeast and in vitro translation systems.^[^ ^13^, ^14^ ^]^ The function of RQC in mammalian physiology and disease remains poorly understood. Listerin has been linked to neurodegenerative diseases in a forward genetic screen study in mice. Listerin homozygous mutant caused profound neurodegeneration in mice with distinct accumulation of soluble hyperphosphorylated Tau.^[^ ^15^ ^]^ Miller et al. subsequently identified a T>C mutation at chr21:28 939 464 in the Listerin gene in AD patients.^[^ ^16^ ^]^ Another tandem mass tag (TMT)‐mass spectrometry (MS) based quantitative proteomics study analyzed 516 dorsolateral prefrontal cortex (DLPFC) tissues from healthy and AD brains.^[^ ^17^ ^]^ The data showed decreased expression of Listerin in the brain tissues of AD patients. These data suggest that Listerin is associated with AD, however, the mechanism by which Listerin is involved in AD is unknown.

In this study, we generated *Listerin* knockout mice to investigate its function in AD. We found that microglia‐specific *Listerin* knockout mice exhibited more severe learning and memory symptoms than those in wild type mice, as well as histological features of neurological disorders. Mechanistically, we found that Listerin has a distinct function in modulating AD via targeting TLR4 signaling to regulate brain inflammation. Listerin is essential for IRE1α‐mediated cleavage and degradation of *TLR4* mRNA, resulting in the downregulated expression of TLR4 protein, which is extremely different from its role that acts as an E3 ubiquitin ligase in the classical RQC pathway. Our present findings imply a possible novel neuroimmune therapy strategy. Targeting Listerin may improve the brain inflammation and symptoms of neuronal decline in Alzheimer's patients. We further clarified the regulatory mechanism of TLR4‐induced brain inflammation in AD pathology, which may provide valuable insights for developing novel therapeutic options for AD in the future.

## 2 Results

### 2.1 Listerin Deletion in Microglia Exacerbates AD Progression In Vivo

**Figure 1:**
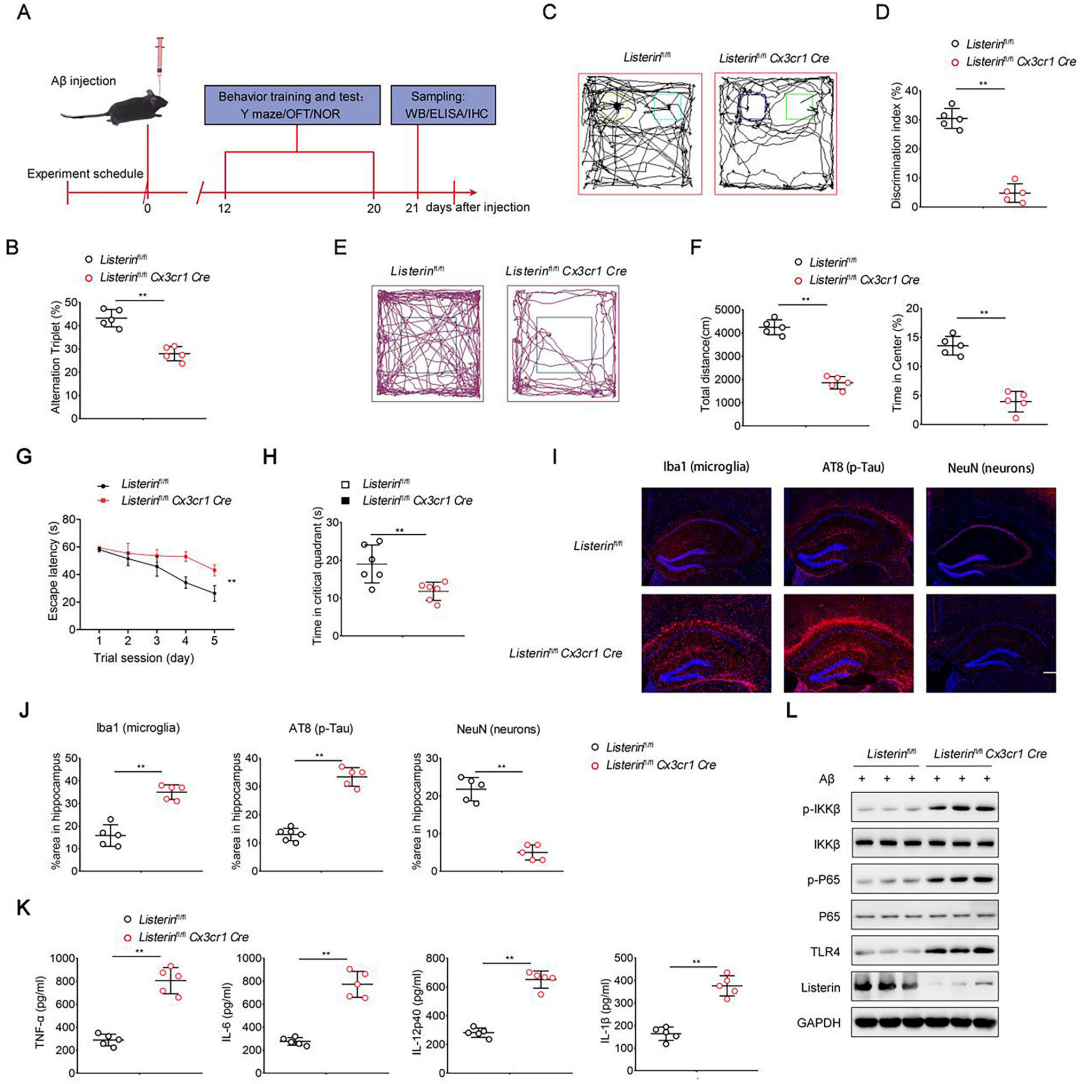
Deletion of *Listerin* aggravate disease in an AD mouse model. A) Schematic diagram of the experimental design. PBS or Aβ were injected into the bilateral entorhinal cortices of 2‐month‐old *Listerin* ^fl/fl^ and *Listerin* ^fl/fl^ *Cx3cr1 Cre* mice. After 12 days, the behavior tests were performed and the sampling were collected at last. B–F) 12 days after Aβ injection in *Listerin* ^fl/fl^ and *Listerin* ^fl/fl^ *Cx3cr1 Cre* mice, Y maze (B), Novel object recognition (C, D) or Open field test (E, F) was performed. G,H) Time to reach hidden platform in Morris water maze and time in critical quadrant (first quadrant) was tested. I,J) Immunolabeling of hippocampi for Iba1 (microglia), AT8 (phospho‐Tau), and NeuN (neurons) of *Listerin* ^fl/fl^ and *Listerin* ^fl/fl^ *Cx3cr1 Cre* mice after Aβ injection. K) TNF‐α, IL‐6, IL‐12p40, and IL‐1β concentrations in the hippocampi of *Listerin* ^fl/fl^ and *Listerin* ^fl/fl^ *Cx3cr1 Cre* mice after Aβ injection measured by ELISA. L) Representative immunoblots of phospho‐IKKβ and phospho‐P65 in the hippocampi of *Listerin* ^fl/fl^ and *Listerin* ^fl/fl^ *Cx3cr1 Cre* mice after Aβ injection. Data shown as mean ± SD and are representative of two independent experiments with similar results. Statistical analysis was performed using unpaired Student's *t‐*test. ^**^ *P* < 0.01.

Mutation of the mouse ortholog *Listerin* leads to neurodegeneration, which suggested that Listern has a crucial function for in this process.^[^ ^15^ ^]^ One large‐scale deep multilayer analysis of brain tissue from AD patients using TMT‐MS‐based quantitative proteomics showed differential expression of Listerin in AD patients compared to healthy individuals (p = 0.000804774).^[^ ^17^ ^]^ We further calculated the mean expression of Listerin and found that the expression of Listerin was lower in the brain tissue of AD patients than in healthy control individuals (Figure S1A, Supporting Information). These data prompted us to explore the role of Listerin in AD. Increasing evidence indicated that microglia‐driven brain inflammation is one of the pivotal characteristics of AD. To evaluate whether *Listerin* deletion in microglia is responsible for brain inflammation and affects the dynamics of AD‐like progression in *vivo*, we generated a mouse model with specific deletion of *Listerin* in primary microglia by mating *Listerin* ^fl/fl^ mice with mice harboring *Cx3cr1 Cre* transgene. Previous studies have shown that the injection of oligomeric Aβ protein into brain can disrupt normal brain function and lead to severe memory deficits, contributing to the cognitive decline observed in AD. Therefore, we simulated the AD model by stereotaxically injecting exogenously synthesized oligomeric Aβ42 protein into the brain of mice.^[^ ^18^ ^]^ We performed the bilateral entorhinal cortices injection of oligomeric Aβ into the *Listerin* ^fl/fl^ and *Listerin* ^fl/fl^ *Cx3cr1 Cre* mice to induced AD model (**Figure** **1**A). The level of damage observed after implementing our model construction was consistent with the results reported in the aforementioned article, compared to the control group.^[^ ^18^, ^19^ ^]^ The Y‐maze test results showed that brain injection of Aβ protein successfully mimicked the behavior of the mouse model of AD, while mice with microglia‐specific depletion of *Listerin* exhibited deteriorative cognitive symptoms upon the extracellular Aβ injection (Figure 1B). Compared with *Listerin* ^fl/fl^ mice, the *Listerin* ^fl/fl^ *Cx3cr1 Cre* mice displayed inferior recognition memory in the novel object recognition test (Figure 1C,D). Deletion of *Listerin* in mice also significantly decreased the traveled distance in an open field test and inhibited their fractional time spent in the center zone (Figure 1E,F). The learning capabilities of mice were tested in Morris water maze to indicate that *Listerin* ^fl/fl^ *Cx3cr1 Cre* mice show severe spatial memory deficits, as investigated in measuring the latency to the platform and time in critical quadrant (Figure 1G,H). Histopathology of the brain showed that *Listerin* ^fl/fl^ *Cx3cr1 Cre* mice displayed redundant Iba1+ microgliosis, increased level of phosphorylated Tau and reductive neurons after injection of Aβ (Figure 1I,J). Notably, microglial‐specific *Listerin* deletion upregulated the expression of inflammatory cytokines in the brain, including TNF‐α, IL‐6, IL‐12, and IL‐1β (Figure 1K). This suggests that Listerin may plays a crucial role in restraining neuroinflammation by modulating cytokine production in microglia. The upregulation of these pro‐inflammatory cytokines indicates that Listerin deficiency may enhance microglial activation, potentially exacerbating neuroinflammatory responses. Given that excessive production of TNF‐α, IL‐6, and IL‐1β has been implicated in neurodegenerative diseases and epilepsy, our findings suggest that Listerin may serve as a protective factor against pathological neuroinflammation. Furthermore, IL‐12 elevation could indicate a shift toward a more pro‐inflammatory microglial phenotype, which may contribute to prolonged neuroinflammation and neuronal dysfunction. These results highlight the importance of Listerin in maintaining microglial homeostasis and suggest that its dysfunction could be a potential driver of neuroinflammatory disorders. Consistently, elevated phosphorylation of IKKβ and P65 were observed in *Listerin* ^fl/fl^ *Cx3cr1 Cre* mice than that in *Listerin* ^fl/fl^ mice (Figure 1L). In addition, we found that the protein levels of TLR4 protein, a classic pattern recognition receptor for lipopolysaccharide (LPS), were signally increased in *Listerin* ^fl/fl^ *Cx3cr1 Cre* mice than that in *Listerin* ^fl/fl^ mice (Figure 1L). To further exclude the effect of the *Cx3cr1 Cre* background, we perform the animal experiments in *Cx3cr1 Cre* and *Listerin* ^fl/fl^ *Cx3cr1 Cre* mice. The results showed that *Listerin* ^fl/fl^ *Cx3cr1 Cre* mice exhibited worse cognitive symptoms and recognition memory upon the extracellular Aβ injection compared with *Cx3cr1 Cre* mice, which was supported by the Y maze test (Figure S1B, Supporting Information) and novel object recognition test (Figure S1C,D, Supporting Information) and open field test (Figure S1E,F, Supporting Information). These data suggested that the effect of Listerin was independent of *Cx3cr1 Cre* background. Altogether, these data suggest that *Listerin* deletion in microglia aggravates the AD progression and inflammation in the brain.

LPS‐induced systemic inflammation and brain inflammation affect the pathological process of AD in various ways.^[^ ^20^ ^]^ In previous studies, an animal model of memory loss similar to Alzheimer's disease was established by injecting LPS into the brain or intraperitoneal cavity of mice.^[^ ^21^ ^]^ To evaluate the role of *Listerin*‐mediated microglial inflammation in AD, we next performed a systemic inflammation model in which LPS was injected into the bilateral entorhinal cortices of the *Listerin* ^fl/fl^ and *Listerin* ^fl/fl^ *Cx3cr1 Cre* mice. Behavioral tests showed that the mice injected with LPS had obvious symptoms of learning and memory impairment related to AD (Figure S2A–E, Supporting Information). The Y‐maze test and the novel object recognition test supported that microglia‐specific depletion of *Listerin* deteriorated cognitive and recognition memory symptoms after extracellular LPS injection (Figure S2A–C, Supporting Information), which was further supported by the open field test (Figure S2D,E, Supporting Information). Depletion of *Listerin* elevated the brain inflammation in the brain after systemic administration of LPS, as evidenced by a significant enhancement in the expression of the proinflammatory cytokines in the hippocampus, such as TNF‐α, IL‐6, IL‐12, and IL‐1β (Figure S2F, Supporting Information). We observed a corresponding increase in the phosphorylation of IKKβ and P65 in the brain in *Listerin* ^fl/fl^ *Cx3cr1 Cre* mice than that in *Listerin* ^fl/fl^ mice (Figure S2G, Supporting Information). There were also higher protein expression levels of TLR4 in *Listerin* ^fl/fl^ *Cx3cr1 Cre* mice than that in *Listerin* ^fl/fl^ mice after LPS stimulation (Figure S2G, Supporting Information). This animal model further suggests that *Listerin* deletion in microglia exacerbates the AD progression and the brain inflammation.

### 2.2 Listerin Inhibits AD‐Related Brain Inflammation

**Figure 2:**
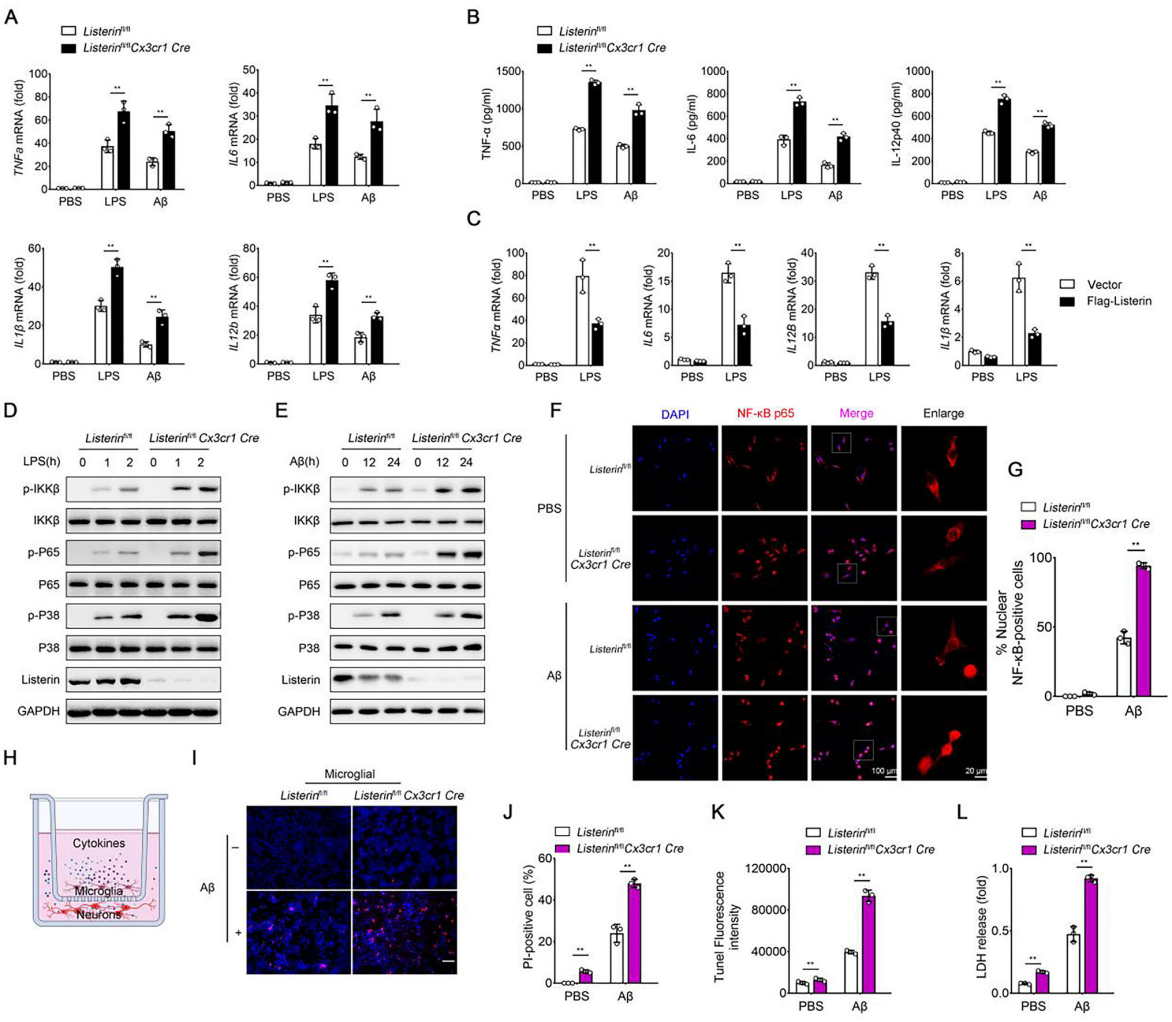
Listerin inhibits AD‐related neuroinflammation. A) Primary microglia prepared from *Listerin* ^fl/fl^ and *Listerin* ^fl/fl^ *Cx3cr1 Cre* mice were stimulated with LPS or Aβ for 24 h, cell lysates were collected and *TNFα*, *IL6*, *IL12b*, and *IL1β* mRNA were quantified by qPCR. B) Supernatant in (A) were collected and TNF‐α, IL‐6 and IL‐12p40 were quantified by ELISA. C) HEK293‐TLR4 cells transfected with Vector or Flag‐Listerin constructs followed by stimulated with LPS for 8 h, cell lysates were collected and *TNFα*, *IL6*, *IL12b*, and *IL1β* mRNA were quantified by qPCR. Bars represent mean ± SD. D) Pathway activation in primary microglia prepared from *Listerin* ^fl/fl^ and *Listerin* ^fl/fl^ *Cx3cr1 Cre* mice under LPS for the indicating times. E) Pathway activation in primary microglia prepared from *Listerin* ^fl/fl^ and *Listerin* ^fl/fl^ *Cx3cr1 Cre* mice under Aβ for the indicating times. F) Confocal images of nuclear translocation of NF‐κB p65 (red) after Aβ treatment in primary microglia prepared from *Listerin* ^fl/fl^ and *Listerin* ^fl/fl^ *Cx3cr1 Cre* mice. G) Quantitative analyses of % nuclear NF‐κB‐positive microglia in (F). H) Schematic representation of co‐culture model: primary microglia prepared from *Listerin* ^fl/fl^ and *Listerin* ^fl/fl^ *Cx3cr1 Cre* mice were plated on transwells. Primary hippocampal neurons from wild type mice were plated in 24‐well plates. I) Representative images of Hoechst and propidium iodide (PI) staining from primary hippocampal neurons co‐incubated with primary microglia prepared from *Listerin* ^fl/fl^ and *Listerin* ^fl/fl^ *Cx3cr1 Cre* mice, and further incubated with Aβ for 3 days. Scale bar, 20 µm. J) Quantification of cell death in (I). K) The cytotoxicity was detected by the TUNEL assay from primary hippocampal neurons co‐incubated with primary microglia prepared from *Listerin* ^fl/fl^ and *Listerin* ^fl/fl^ *Cx3cr1 Cre* mice, and further incubated with Aβ for 3 days. L) LDH assay from primary hippocampal neurons co‐incubated with primary microglia prepared from *Listerin* ^fl/fl^ and *Listerin* ^fl/fl^ *Cx3cr1 Cre* mice, and further incubated with Aβ for 3 days. Data shown as mean ± SD and are representative of two independent experiments with similar results. Statistical analysis was performed using unpaired Student's *t‐*test. ^**^ *P* < 0.01.

To ascertain the involvement of *Listerin* in the establishment and progression of brain inflammation, we first performed the knockdown assay in mouse cell lines of glial cells (BV2). The results showed that knockdown of Listerin upregulated the expression of TNF‐α, IL‐6, IL‐12, and IL‐1β in BV2 cells (Figure S3A,B, Supporting Information) after the LPS or Aβ stimulation. These results preliminarily indicate that knockdown of Listerin promotes AD‐related inflammation. To further confirm the role of Listerin in AD‐related brain inflammation, we performed experiments in primary microglia from newborn mouse pups. We found that primary microglia from *Listerin* ^fl/fl^ *Cx3cr1 Cre* mice showed significantly increased LPS‐ or Aβ‐induced *TNFα*, *IL6*, *IL12b*, and *IL1β* mRNA transcription levels compared with those from *Listerin* ^fl/fl^ mice (**Figure** **2**A). Higher TNF‐α, IL‐6, and IL‐12 secretion were measured in primary microglia from *Listerin* ^fl/fl^ *Cx3cr1 Cre* mice compared with that from the *Listerin* ^fl/fl^ mice (Figure 2B). Similarly, Aβ‐induced mRNA levels and protein secretion of TNF‐α, IL‐6, and IL‐12 was evaluated in primary microglia from *Listerin* ^fl/fl^ *Cx3cr1 Cre* mice compared with that from the *Cx3cr1 Cre* mice (Figure S1G,H, Supporting Information). In contrast, overexpression of Listerin in HEK293 cells stably expressing TLR4 cells (HEK293‐TLR4 cells) attenuated LPS‐induced *TNFα*, *IL6*, *IL12b*, and *IL1β* mRNA expression (Figure 2C).

Since TLR‐mediated activation of nuclear factor κB (NF‐κB) and MAPKs lead to the production of inflammatory cytokines, we next explored the role of Listerin in the NF‐κB and MAPKs signaling pathways. We found that knockout of *Listerin* in primary microglia could enhance LPS‐ and Aβ‐induced phosphorylation of IKKβ, P65, and P38 (Figure 2D,E). We further assessed the nuclear translocation of NF‐κB, we found that knockdown of *Listerin* promoted the nuclear translocation of NF‐κB in BV2 cells (Figure S3C,D, Supporting Information). We further found that the nuclear translocation of NF‐κB in *Listerin* ^fl/fl^ *Cx3cr1 Cre* primary microglia showed at least a 2‐fold increase compared with that in *Listerin* ^fl/fl^ primary microglia after stimulation with Aβ (Figure 2F,G).

Microglia‐mediated brain inflammation plays an important role in the pathogenesis of AD, which can destroy the structure and function of neurons, leading to neuronal degeneration and death.^[^ ^22^ ^]^ Thus, we evaluated whether *Listerin* knockout microglia were more neurotoxic than *Listerin* WT microglia. WT neurons were co‐cultured with microglia in a noncontact transwell system, which allows for the exchange of soluble cytokines (Figure 2H). Microglia treated with Aβ induced neuronal cell death as measured by Hoechst/PI staining. Notably, compared to Aβ‐treated WT microglia, Aβ‐treated Listerin knockout microglia induced more neuronal cell death (Figure 2I,J). Moreover, we further showed that *Listerin*‐knockout microglia treated with Aβ showed more potent neurotoxicity on neuronal cells compared to *Listerin* WT microglia through TUNEL staining and LDH assay (Figure 2K,L). These data indicate that Listerin may inhibit AD‐related brain inflammation, thereby alleviating Aβ‐induced neurotoxicity.

### 2.3 Listerin Decreases the Expression of TLR4 Protein

**Figure 3:**
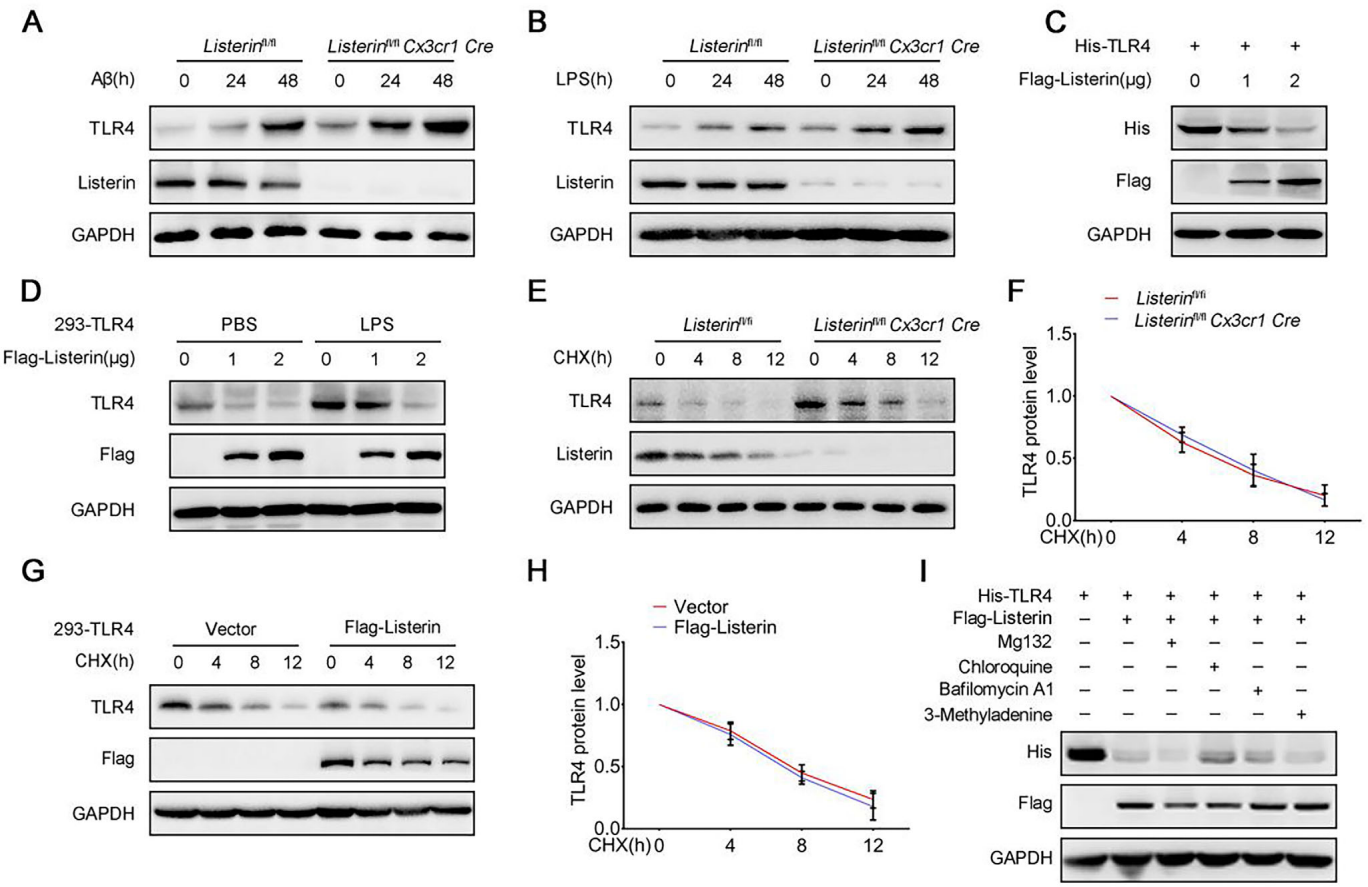
Listerin decreases the expression of TLR4 protein. A) Representative immunoblots of primary microglia prepared from *Listerin* ^fl/fl^ and *Listerin* ^fl/fl^ *Cx3cr1 Cre* mice under LPS stimulation for the indicating times. B) Representative immunoblots of primary microglia prepared from *Listerin* ^fl/fl^ and *Listerin* ^fl/fl^ *Cx3cr1 Cre* mice under Aβ stimulation for the indicating times. C) Representative immunoblots of HEK293T cells transfected with His‐TLR4 together with gradient amount of Flag‐Listerin constructs. D) Representative immunoblots of HEK293‐TLR4 cells transfected with gradient amount of Flag‐Listerin constructs and stimulated with LPS for 8 h. E) Representative immunoblots of primary microglia prepared from *Listerin* ^fl/fl^ and *Listerin* ^fl/fl^ *Cx3cr1 Cre* mice followed by stimulated with LPS for 4 h and then treated with the protein synthesis inhibitor CHX for the indicated times. F) Quantification analysis of proteins degradation kinetics in (E). G) Representative immunoblots of HEK293‐TLR4 cells transfected with Vector or Flag‐Listerin constructs followed by stimulated with LPS for 4 h and then treated with the protein synthesis inhibitor CHX for the indicated times. H) Quantification analysis of proteins degradation kinetics in (G). I) Representative immunoblots of HEK293T cells transfected with Vector or Flag‐Listerin constructs followed by treatment with Mg132 (10 µm), Chloroquine (10 µm), Bafilomycin A1 (0.5 µm) or 3‐Methyladenine (10 mm) for 10 h.

Up‐regulated TLR4 in microglial cells can lead to the hyperactivation of neuroinflammatory, neuronal degeneration, and further aggravate the progression of AD.^[^ ^23^ ^]^ In our mouse models, we found increased protein expression levels of TLR4 in *Listerin* ^fl/fl^ *Cx3cr1 Cre* mice than that in *Listerin* ^fl/fl^ mice with Aβ or LPS stimulation (Figure 1J; Figure S2G, Supporting Information), indicating that Listerin may target TLR4 to participate in brain inflammation. We further confirmed that the deficiency of Listerin increased the TLR4 protein level in primary microglia from Listerin^fl/fl^ and Listerin^fl/fl^ *Cx3cr1 Cre* mice after LPS or Aβ treatment (**Figure** **3**A,B). In contrast, we found that overexpression of Listerin attenuated TLR4 protein expression in a dose‐dependent manner in HEK293T cells (Figure 3C). Similarly, we detected that LPS‐induced TLR4 protein expression was declined by Listerin in HEK293‐TLR4 cells (Figure 3D).

To explore the mechanism of Listerin‐induced down‐regulation of TLR4, we first performed a cycloheximide (CHX) assay to study the degradation rate of the TLR4 protein. Consistently, we found that the level of TLR4 protein was greater in *Listerin* ^fl/fl^ *Cx3cr1 Cre* primary microglia compared to that in *Listerin* ^fl/fl^ primary microglia. Intriguingly, the turnover rate of the TLR4 protein was not significantly different between *Listerin* ^fl/fl^ *Cx3cr1 Cre* microglia and *Listerin* ^fl/fl^ microglia after treatment with CHX both at steady state (t = 0) and at various times (Figure 3E,F). In contrast, overexpression of Listerin decreased the level of TLR4 protein in HEK293‐TLR4 cells (Figure 3G), while the turnover rate of TLR4 protein remained unchanged after exposure with CHX (Figure 3H). Thus, Listerin does not regulate TLR4 protein stability to downregulate its protein level. The Ubiquitin‐proteasome pathway and autophagy‐lysosomal pathway are two major pathways of protein degradation.^[^ ^24^ ^]^ To further confirm that Listerin does not regulate TLR4 protein stability, we tested whether inhibitors of the proteasome (Mg132) or autophagy (Chloroquine, Bafilomycin A1, 3‐Methyladenine) could block the TLR4 protein downregulation. We found that Listerin‐induced TLR4 attenuation could not be reversed by these inhibitors (Figure 3I), indicating that the proteasome and autophagy pathways were not involved in the Listerin‐induced downregulation of TLR4 protein. Together, these data suggest that Listerin regulates TLR4 protein expression but not through the protein degradation.

### 2.4 Listerin Decreased TLR4 mRNA Transcript

**Figure 4:**
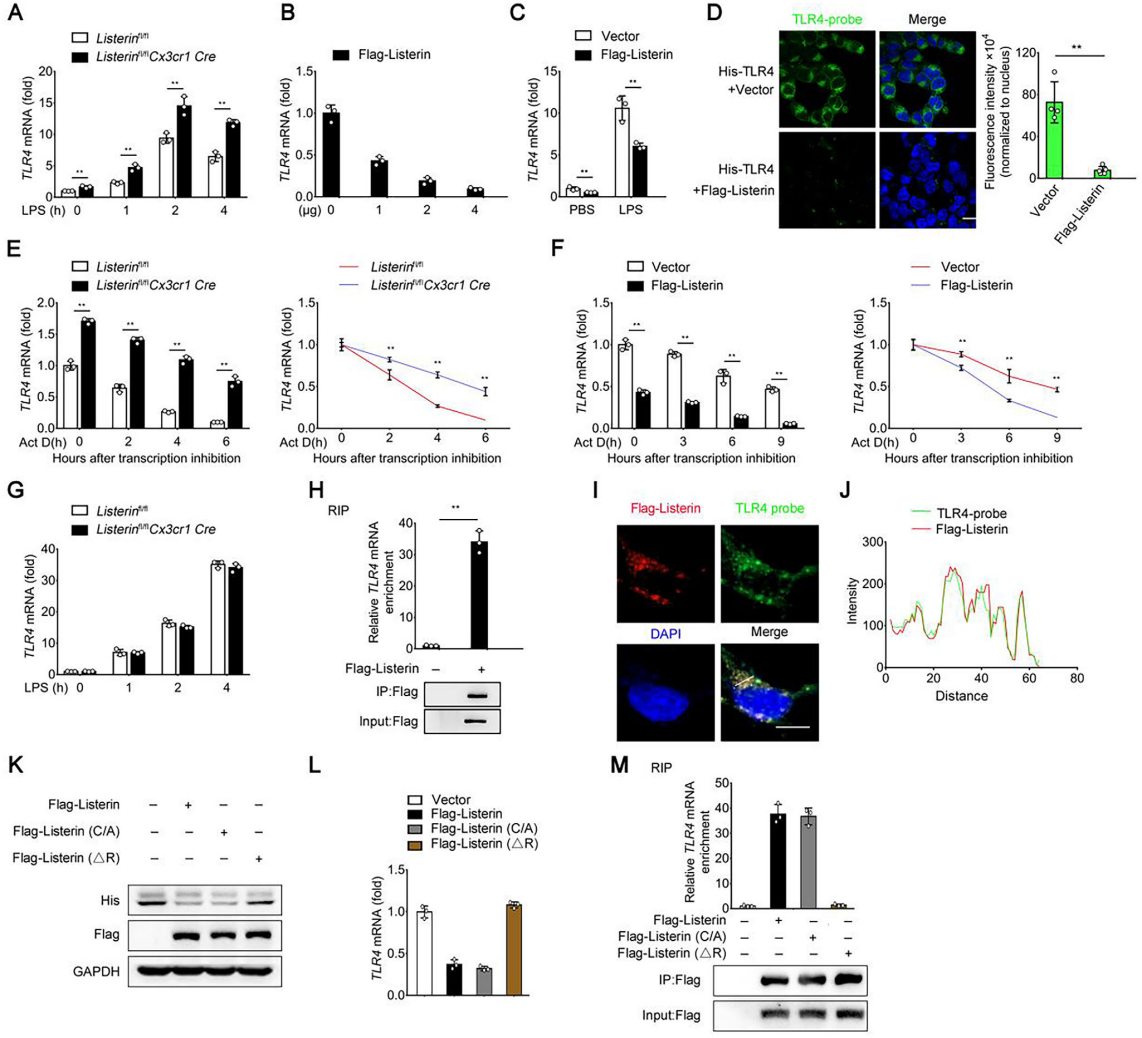
Listerin decreased *TLR4* mRNA transcript levels. A) Primary microglia prepared from *Listerin* ^fl/fl^ and *Listerin* ^fl/fl^ *Cx3cr1 Cre* mice were stimulated with LPS for 0–4 h, cell lysates were collected and *TLR4* mRNA was quantified by qPCR. B) HEK293T cells transfected with His‐TLR4 together with gradient amount of Flag‐Listerin constructs, cell lysates were collected and *TLR4* mRNA was quantified by qPCR. C) HEK293‐TLR4 cells transfected with Vector or Flag‐Listerin constructs followed by stimulated with PBS or LPS for 4 h, cell lysates were collected and *TLR4* mRNA was quantified by qPCR. D) Representative images of FISH for *TLR4* mRNA in HEK293T cells transfected with His‐TLR4 together with Vector or Flag‐Listerin constructs. Images are representative of 3 independent experiments. Scale bars, 20 µm. E) Primary microglia prepared from *Listerin* ^fl/fl^ and *Listerin* ^fl/fl^ *Cx3cr1 Cre* mice were stimulated with LPS for 4 h and then treated with actinomycin D for the indicated times, cell lysates were collected and *TLR4* mRNA (left) and *TLR4* mRNA degradation (right) was quantified by qPCR. F) HEK293‐TLR4 cells were transfected with Vector or Flag‐Listerin constructs followed by stimulated with LPS for 4 h and then treated with actinomycin D for the indicated times, cell lysates were collected and *TLR4* mRNA (left) and *TLR4* mRNA degradation (right) was quantified by qPCR. G) Primary microglia prepared from *Listerin* ^fl/fl^ and *Listerin* ^fl/fl^ *Cx3cr1 Cre* mice were stimulated with LPS for 4 h and then exposed to a 30‐min EU pulse, nascent RNA was extracted and *TLR4* mRNA was quantified by qPCR. H) RIP assay for *TLR4* mRNA in HEK293‐TLR4 cells transfected with Vector or Flag‐Listerin constructs. Normalized data were shown as relative fold enrichment to the control group. I) Representative images of FISH for *TLR4* mRNA and fluorescence immunostaining for Flag‐Listerin in HEK293T cells transfected with His‐TLR4 together with Flag‐Listerin constructs. J) Intensity profiles in (I). K) Representative immunoblots of HEK293‐TLR4 cells transfected with vector, Flag‐Listerin, Flag‐Listerin (C/A) or Flag‐Listerin (△R) constructs. Results are representative of 3 independent experiments. L) Cells treated as in (K) were collected and *TLR4* mRNA were quantified by qPCR. M) Representative immunoprecipitation (IP) of HEK293T cells transfected with Myc‐IRE1α together with vector, Flag‐Listerin, Flag‐Listerin (C/A) or Flag‐Listerin (△R) constructs. Data shown as mean ± SD and are representative of three independent experiments with similar results. Statistical analysis was performed using unpaired Student's *t‐*test. ^**^ *P* < 0.01.

The protein content in cells is mediated by both transcription and translation. We next turned to detect the expression of *TLR4* mRNA. The *TLR4* mRNA levels increased in response to LPS, which were further increased in *Listerin* ^fl/fl^ *Cx3cr1 Cre* primary microglia compared to that in *Listerin* ^fl/fl^ primary microglia (**Figure** **4**A). Listerin overexpression decreased the *TLR4* mRNA level in a dose‐dependent manner in HEK293‐TLR4 cells (Figure 4B). We further found that Listerin overexpression attenuated the *TLR4* mRNA level in HEK293‐TLR4 cells stimulated with LPS (Figure 4C). In addition, we synthesized the fluorescence labeled probe of *TLR4* mRNA to visually detect the RNA in cells. Overexpression of Listerin diminished the fluorescence of TLR4 probe by in situ hybridization (FISH) assay (Figure 4D). To more precisely understand how Listerin regulates *TLR4* mRNA levels, we sought to reveal the underlying regulatory mechanisms. The total RNA level in cells is maintained by both mRNA biogenesis and decay. To analyze whether Listerin increases the decay of *TLR4* transcripts, we conducted RNA stability assays by the addition of Actinomycin D (Act D), which can inhibit RNA synthesis by intercalating into DNA and preventing transcription to assess the stability and degradation dynamics of specific mRNA transcripts. We found that knockout of *Listerin* prolonged the half‐life of *TLR4* mRNA in primary microglia (Figure 4E). On the contrary, forced expression of Listerin shortened the half‐life of *TLR4* mRNA in HEK293T cells (Figure 4F). We further investigated whether Listerin regulates *TLR4* mRNA biogenesis by segregating nascent RNA through metabolic pulse labeling with 5‐ethyluridine (EU). The result showed the same production of EU‐labeled *TLR4* mRNA in primary microglia from *Listerin*‐depleted *Listerin* ^fl/fl^ *Cx3cr1 Cre* mice and *Listerin* ^fl/fl^ primary microglia, indicating that *Listerin* has no effect on the kinetics of *TLR4* mRNA biogenesis (Figure 4G).

To further prove that Listerin targets *TLR4* mRNA, we investigated the interaction between Listerin and *TLR4* mRNA. We performed the RNA binding protein immunoprecipitation assay (RIP) and found that Listerin interacted with *TLR4* mRNA (Figure 4H), but not with *TLR2*, *TLR3*, or *TLR9* mRNA (Figure S4A–C, Supporting Information), which illustrates that Listerin specifically binds to *TLR4* mRNA. We further cultured BV2 cells and mouse primary microglia cells for RIP assay. The results showed that Listerin interacted with *TLR4* mRNA in BV2 (Figure S4D, Supporting Information) and primary microglia cells (Figure S4E, Supporting Information), and this interaction was enhanced with Aβ stimulation. These data show that Listerin directly acts on *TLR4* mRNA in the microglia at physiological and pathological conditions. In addition, confocal imaging by FISH assay confirmed the colocalization of Listerin and *TLR4* mRNA (Figure 4I,J). In order to analyze the interactions between Listerin and *TLR4* mRNA, we used the catRAPID omics algorithm (http://service.tartaglialab.com/page/catrapid_group) to predicate their interaction. The analysis revealed that the TLR4 mRNA sequence spanning 2301–2452 nucleotides exhibited the highest interaction propensity with Listerin. To confirm this prediction, we constructed six truncated fragments of *TLR4* mRNA covering its full length: Fragment 1: 1–500 nt;Fragment 2: 501–1000 nt;Fragment 3: 1001–1500 nt;Fragment 4: 1501–2000 nt;Fragment 5: 2001–2300 nt;Fragment 6: 2301–2452 nt. RNA pull‐down assays demonstrated that only Fragment 6 (2301‐2452 nt) showed strong binding to Listerin protein, validating the computational prediction (Figure S4F, Supporting Information).

Listerin is a RING type E3 ubiquitin ligase. To verify whether the E3 ubiquitin ligase activity of Listerin contributes to the regulation of TLR4, we next constructed two Listerin mutants that were deprived of the potential E3 enzymatic activity, ‐Listerin (ΔR) in which the putative C‐terminal RING domain was deleted, and ‐Listerin (C/A) in which contains point mutations in the RING domain. Deletion of the RING domain did not attenuate the expression of TLR4 protein and mRNA level mediated by Listerin in HEK293‐TLR4 cells. However, point mutation Listerin (C/A) still had the ability to reduce TLR4 expression (Figure 4K,L), indicating that the RING domain of Listerin is essential for the reduction of TLR4 but the E3 ligase activity of Listerin was not required. We found that full length Listerin and Listerin (C/A) rather than Listerin (ΔR) interacted with *TLR4* mRNA in the RIP assay (Figure 4M), suggesting that the RING domain of Listerin was essential for the interaction with *TLR4* mRNA. In conclusion, these data demonstrated that Listerin binds to *TLR4* mRNA and decreases its mRNA stability and accumulation.

### 2.5 Listerin is Essential for IRE1α to Induce TLR4 mRNA Decay

**Figure 5:**
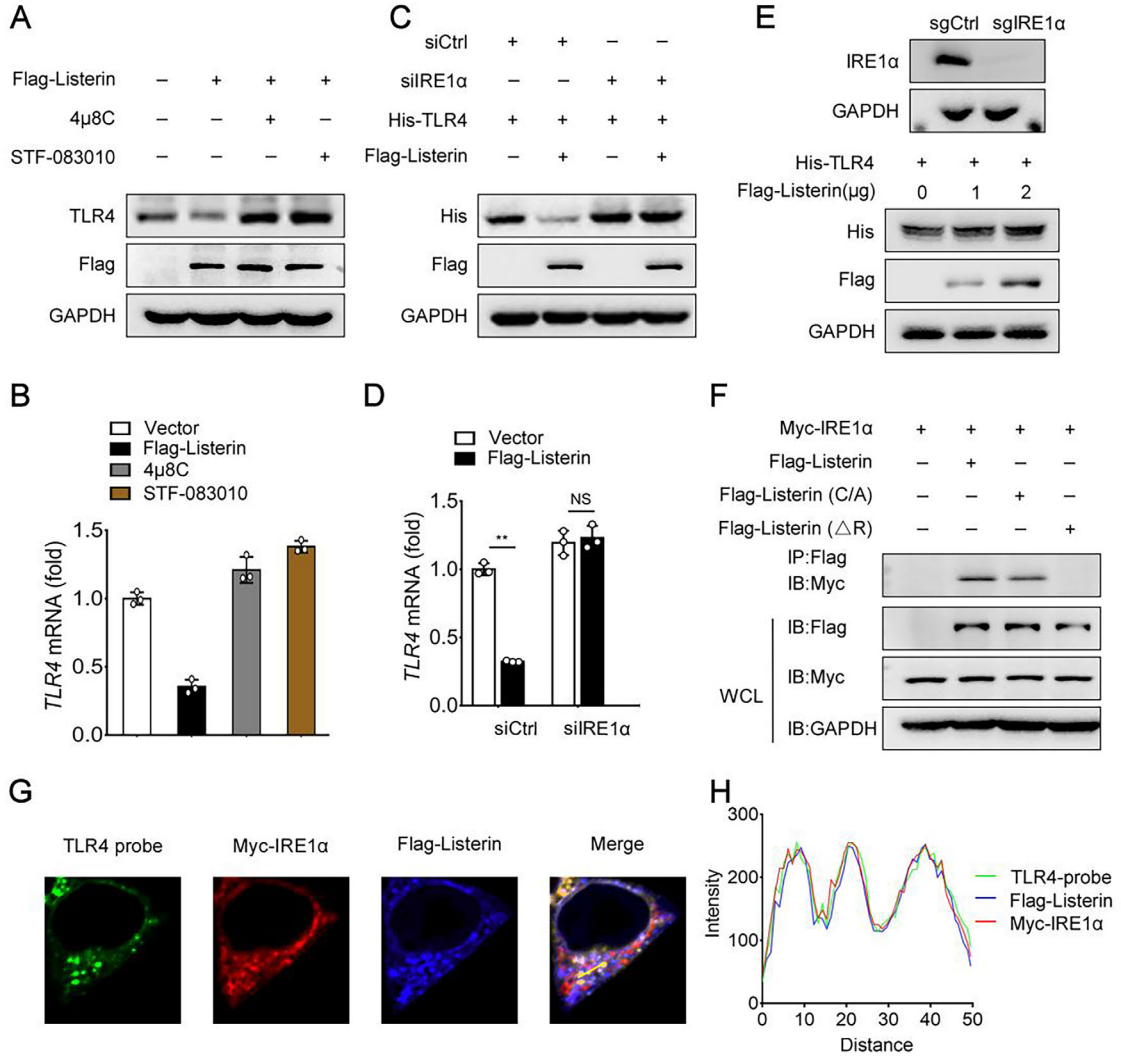
IRE1α is required for Listerin mediated TLR4 degradation. A) Representative immunoblots of HEK293‐TLR4 cells transfected with vector or Flag‐Listerin constructs for 20 h and then treated with 4µ8C (10 µm) or STF‐083010 (10 µm) for 10 h. B) Cells treated as in (A) were collected and *TLR4* mRNA were quantified by qPCR. C) Representative immunoblots of HEK293T cells silenced for control (siCtrl) or IRE1α (siIRE1α) for 24 h followed by transfected with His‐TLR4 together with vector or Flag‐Listerin constructs for 24 h. D) Cells treated as in (C) were collected and *TLR4* mRNA were quantified by qPCR. E) Representative immunoblots of IRE1α knockout cells transfected with His‐TLR4 together with gradient amount of Flag‐Listerin constructs. F) Co‐IP analysis of the interaction in HEK293‐TLR4 cells transfected with Myc‐IRE1α together with vector, Flag‐Listerin, Flag‐Listerin (C/A) or Flag‐Listerin (△R) constructs. Normalized data were shown as relative fold enrichment to the control group. G) Representative images of FISH for *TLR4* mRNA and fluorescence immunostaining for Flag‐Listerin and Myc‐IRE1α in HEK293T cells transfected with His‐TLR4, Myc‐IRE1α together with Flag‐Listerin constructs. H) Intensity profiles in (G).Data shown as mean ± SD and are representative of three independent experiments with similar results. Statistical analysis was performed using unpaired Student's *t‐*test. ^**^ *P* < 0.01.

Endoplasmic Reticulum stress has been reported to be closely associated with neurodegenerative diseases with protein aggregates pathology, such as AD, Frontotemporal dementia (FTD), Parkinson's disease (PD), Huntington disease and Amyotrophic lateral sclerosis (ALS).^[^ ^25^ ^]^ IRE1 α is the most evolutionarily conserved UPR sensor in endoplasmic reticulum stress response, targeting not only the classical XBP‐1 mRNA but also other specific mRNAs, mediating a process called RIDD.^[^ ^11^, ^26^ ^]^ ER stress has been shown to be involved in the pathogenesis of AD, Aβ treatment can activate the ER stress.^[^ ^27^ ^]^ Therefore, we next tested whether IRE1α has effects on the *TLR4* mRNA degradation mediated by Listerin. To this end, we employed two inhibitors in our study, 4µ8C (a specific inhibitor of IRE1α RNase) and STF‐083010 (a small‐molecule inhibitor of IRE1α that restrains the endonuclease activity of IRE1α).^[^ ^28^ ^]^ We found that these two inhibitors successfully blocked the decrease of TLR4 protein and mRNA level mediated by Listerin overexpression in HEK293‐TLR4 cells (**Figure** **5**A,B) and in HEK293T cells (Figure S5A,B, Supporting Information). siRNA‐mediated endogenous IRE1α knockdown in HEK293T cells reversed the reduction of TLR4 protein and mRNA by Listerin (Figure 5C,D). We further used CRISPR/Cas9 gene targeting to generate IRE1α knockout HEK293T cells and found that Listerin did not attenuate TLR4 protein expression in IRE1α KO cells (Figure 5E). These data suggest that IRE1α is required for the Listerin‐mediated reduction of TLR4 expression. We further showed that IRE1α interacted with full length Listerin and Listerin (C/A), but not with Listerin (ΔR) by immunoprecipitation (IP) assys in HEK293T cells (Figure 5F). These data suggest that Listerin binds to IRE1α through its RING domain to cleave *TLR4* mRNA. Indeed, we showed that Listerin distinctly colocalized with *TLR4* mRNA and IRE1α. While, IRE1α displayed colocalization with *TLR4* mRNA only in the presence of Listerin (Figure 5G,H; Figure S5C–F, Supporting Information). All together, these data manifest that Listerin may act as a crucial scaffold protein supporting IRE1α to cleavage *TLR4* mRNA through its RING domain.

### 2.6 IRE1α Cleaves *TLR4* mRNA through RIDD

**Figure 6:**
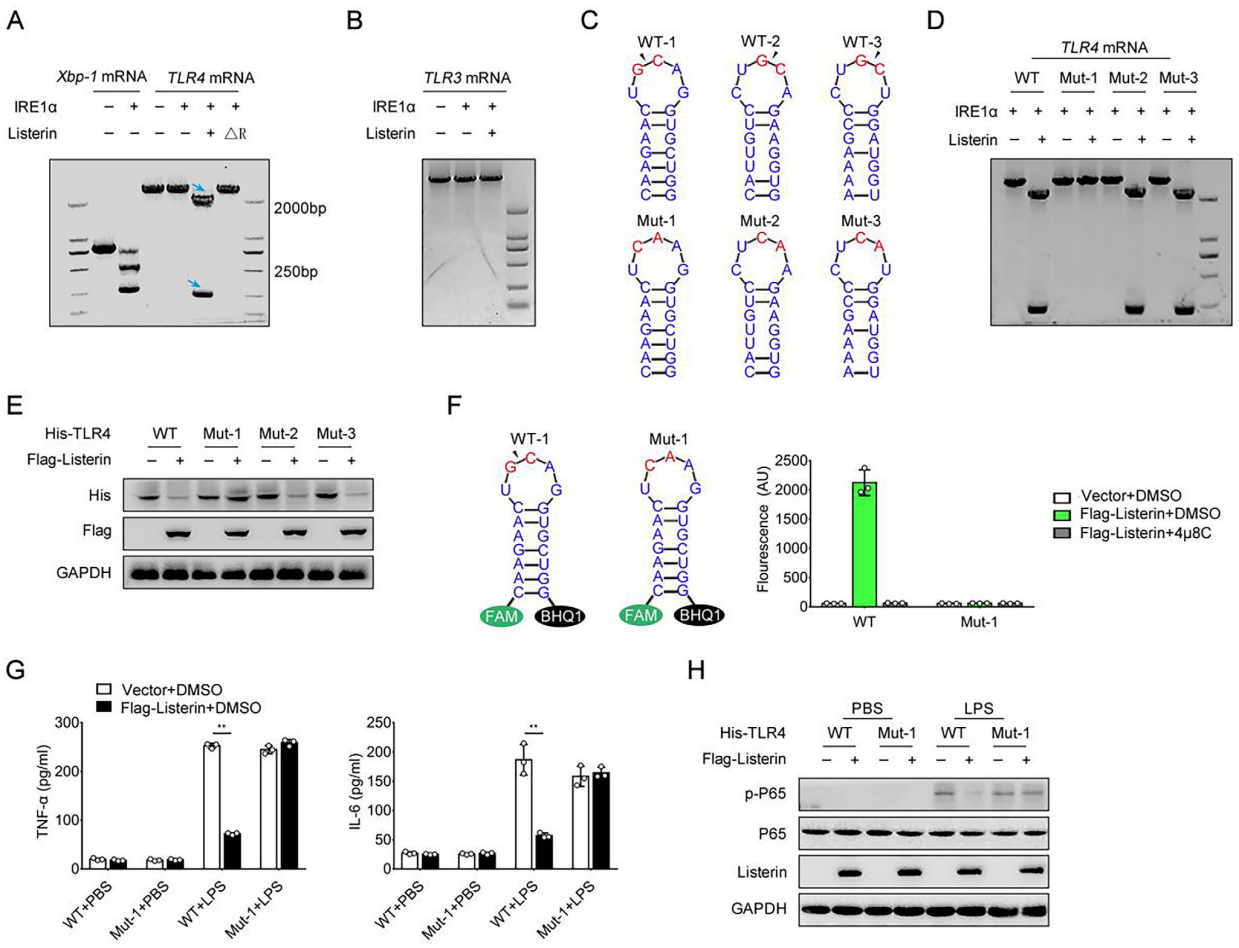
Listerin is essential for IRE1α to cleavage *TLR4* mRNA. A) RNA cleavage assay of *Xbp‐1* or *TLR4* transcripts incubated with recombinant protein IRE1α together with PBS, Listerin or Listerin (△R) for 30 min, followed by resolved for a 2.5% agarose gel and visualized by a BioRad Molecular Imager. B) RNA cleavage assay of *TLR3* transcripts incubated with recombinant protein IRE1α together with PBS or Listerin for 30 min, followed by resolved for a 2.5% agarose gel and visualized by a BioRad Molecular Imager. C) Illustration of predicted stem‐loop secondary structure shown for human wild type (WT) *TLR4* mRNA and the mutant (Mut, GC to CA) *TLR4* mRNA. The potential IRE1α cleavage site is indicated by an arrow. D) RNA cleavage assay of WT or Mut *TLR4* transcripts incubated with recombinant protein IRE1α together with PBS or Listerin for 30 min, followed by resolved for a 2.5% agarose gel and visualized by a BioRad Molecular Imager. E) Representative immunoblots of HEK293T cells transfected with WT or Mut His‐TLR4 together with vector or Flag‐Listerin constructs. F) Fluorescence‐based analysis of IRE1α cleavage of the synthetic WT‐1 or mutant (mut, GC to CA) Mut‐1 mRNA. G) HEK293T cells were transfected with WT or Mut‐1 His‐TLR4 together vector or Flag‐Listerin constructs followed by stimulated with LPS for 6 h, cell supernatant was collected and TNF‐α (left) or IL‐6 (right) were quantified by ELISA. H) Representative immunoblots of phospho‐P65 in HEK293T cells treated as in (G). Data shown as mean ± SD and are representative of three independent experiments with similar results. Statistical analysis was performed using unpaired Student's *t‐*test. ^**^ *P* < 0.01.

The data described above indicated that IRE1α is required for Listerin‐mediated *TLR4* mRNA decay. IRE1α, through its RNase activity, can initiate a direct cleavage reaction and thus affect the stability of multiple RNAs.^[^ ^29^ ^]^ We next investigated how IRE1α cleaves *TLR4* mRNA. As reported, the in vitro RNA cleavage assay showed that recombinant IRE1α efficiently cleaved Xbp‐1. However, IRE1α could not cleave *TLR4* mRNA directly. IRE1α cleaved the *TLR4* mRNA at a single site in the presence of full‐length Listerin, but not Listerin (ΔR) (**Figure** **6**A). These data are consistent with our above data that Listerin is important for IRE1α‐mediated cleavage of *TLR4* mRNA. We further detected that IRE1α could not cleave the *TLR3* mRNA in the presence of full‐length Listerin, which suggested the cleavage effect of Listerin on *TLR4* mRNA is specific (Figure 6B). To confirm whether *TLR4* mRNA acts a potent candidate for RIDD substrate, we carried out a bioinformatic analysis combined with predictions of mRNA secondary structure. We found that *TLR4* mRNA contains three putative XBP1‐like stem‐loop endomotifs, and possesses the core postulated RIDD consensus sequence CNG|CAGN within the predicted stem loop secondary structures (Figure 6C). To confirm the effect of IRE1α at the putative cleavage sites, we generated three synthetic mutant *TLR4* mRNA substrates (Mut‐1, Mut‐2, Mut‐3) according to the bioinformatic analysis, in which we replaced the conserved GC with CA in the loop to entirely abolish the single‐site cleavage site (Figure 6C). The in vitro cleavage assay showed that the mutation of the stem‐loop structure of the *TLR4* mRNA at site 1 (Mut‐1) abrogated its effect to be cleaved by IRE1α protein in the presence of Listerin (Figure 6D). The 5′ or 3′ fragments of *TLR4* mRNA produced by IRE1α agreed in size with this location of the stem‐loop motifs, which indicated that IRE1 most probably cleaves *TLR4* mRNA at the WT‐1 site in the presence of Listerin. Consistent with the in vitro cleavage data, we found that overexpression of Listerin decreased the protein expression of TLR4 transfected with His‐TLR4 WT, Mut‐2 and Mut‐3, but not Mut‐1 in HEK293T cells (Figure 6E).

We further synthetized the 19‐nucleotide *TLR4* mRNA fragments labeled with the 5′‐FAM fluorophore and 3′‐black hole quencher agent (BHQ) as the substrate for in vitro biochemical assays to assess whether this stem‐loop of *TLR4* mRNA can be splited by purified recombinant IRE1α protein (Figure 6F). The strong fluorescence signal in this assay indicated that only the addition of Listerin promotes *TLR4* mRNA cleavage by IRE1α, which can be completely abolished by 4µ8C. Simultaneously, the GC‐to‐CA mutant *TLR4* mRNA (Mut‐1) entirely canceled the cleavage‐based fluorescence signal compared with its WT fragment, which confirmed the function of IRE1α at the predictive cleavage site (Figure 6F). Consistent with the in vitro cleavage data, LPS‐induced production of TNF‐α or IL‐6 and phosphorylation of P65 were inhibited by Listerin in His‐TLR4 WT transfected HEK293T cells, while Listerin did not have this effect in HEK293T cells transfected with His‐TLR4 Mut‐1 (Figure 6G,H). All together, these results indicate that *TLR4* mRNA is an RIDD substrate for IRE1α and Listerin is required for the cleavage of *TLR4* mRNA mediated by IRE1α.

### 2.7 Listerin Ameliorates the Inflammation of AD Progression In Vivo

**Figure 7:**
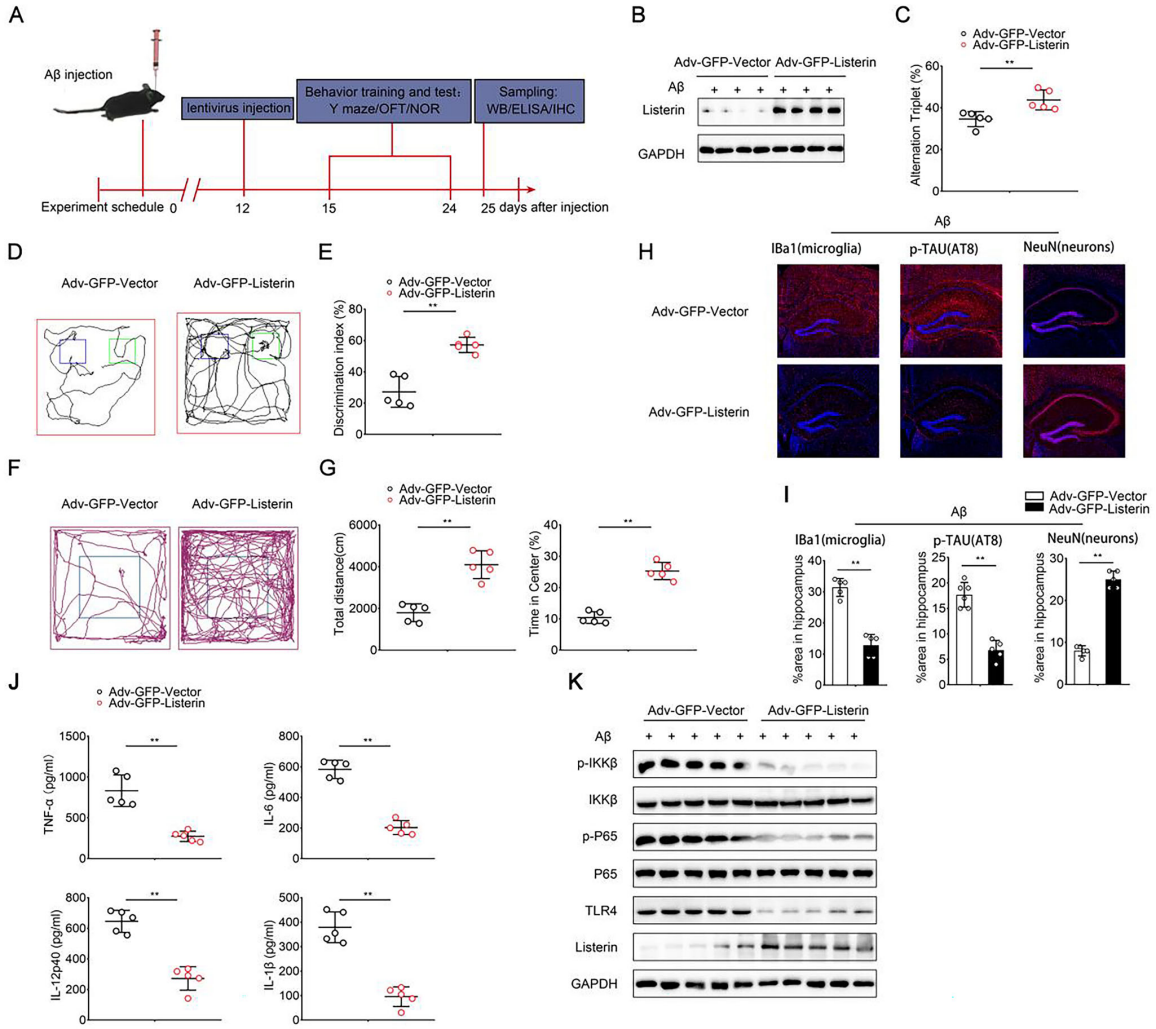
Listerin ameliorates the inflammation of AD progression in vivo. A) Schematic diagram of the experimental design. Aβ were injected into the bilateral entorhinal cortices of 2‐month‐old WT mice. After 12 days, Adv‐GFP or Adv‐Listerin‐GFP were injected into the bilateral entorhinal cortices for 3 days, the behavior tests were performed and the sampling were collected at last. B) Representative immunoblots of Listerin expression in hippocampi from mice treated in (A). Results are representative of 3 mice. C–G) Mice were treated as in (A), Y maze (C), Novel object recognition (D, E) or Open field test (F, G) was performed (n = 5 mice per group). H) Immunolabeling of hippocampi for Iba1 (microglia), AT8 (phospho‐Tau), and NeuN (neurons) of mice treated as in (A). I) TNF‐α, IL‐6, IL‐12p40, and IL‐1β concentrations in the hippocampi of mice treated as in (A) measured by ELISA (n = 5 mice per group). J) Representative immunoblots of phospho‐IKKβ and phospho‐P65 in the hippocampi of mice treated as in (A). Data shown as mean ± SD and are representative of three independent experiments with similar results. Statistical analysis was performed using unpaired Student's *t‐*test. ^**^ *P* < 0.01.

To determine the therapeutic potential of Listerin, adenovirus‐mediated overexpression of Listerin (Adv‐GFP‐Listerin) was used in the Aβ induced‐AD model (**Figure** **7**A). The efficiency of Adv‐GFP‐Listerin was validated using western blot (Figure 7B). Then, we proceeded to test the therapeutic potential of Listerin in vivo using the Aβ‐induced mouse model of AD. The Y‐maze test showed that mice with injection of Adv‐GFP‐Listerin ameliorated cognitive symptoms upon the extracellular Aβ injection (Figure 7C). Stereotaxic injection of Listerin improved the recognition memory impairment of mice in the novel object recognition test (Figure 7D,E). We also found that the mice with Listerin injection traveled longer distances in the open field test and spent more their fractional time in the center zone (Figure 7F,G). The results of these behavioral tests suggested that lentivirus‐mediated overexpression of Listerin improved the recognition memory of Aβ‐induced mouse model of AD. Furthermore, pathological features in brain showed less Iba1^+^ microgliosis, reduced levels of phosphorylated Tau and an increased number of neurons after Adv‐GFP‐Listerin injection (Figure 7H,I). Inflammatory cytokines in the brain were also reduced after the Adv‐GFP‐Listerin injection (Figure 7J). We also detected the decreased phosphorylation of IKKβ and P65 in Adv‐GFP‐Listerin injected mice compared with Adv‐GFP‐Vector injected mice (Figure 7K). Together, these data indicate that Listerin overexpression decelerates the disease progression in a mouse model of Aβ‐mediated AD.

## 3 Discussion

Listerin is best known for its primary roles in the RQC system. Aberrant and misfolded polypeptide nascent chains can undergo modification with polyubiquitin by Listerin, thereby initiating aberrant nascent‐chain with ubiquitin for proteasome‐mediated degradation.^[^ ^13b,c^ ^]^ Earlier studies have shown the embryonic lethality and neurodegenerative phenotypes of Listerin‐mutant mice. A mutation in the mouse Listerin homolog, lister, results in a neurodegenerative disorder with phosphorylated Tau accumulation.^[^ ^15^ ^]^ However, the underlying mechanism remains unclear. In this study, we show that Listerin regulates brain inflammation and plays a unique regulatory role in AD that is independent of its classical function in RQC. Listerin is a scaffold protein that supports IRE1α to promote the cleavage and degradation of *TLR4* mRNA, which leads to impaired TLR4 protein expression and is quite different from its role in the classical RQC pathway. Importantly, we found that Listerin promoted the degradation of *TLR4* transcripts rather than TLR4 protein, which is different from its function in RQC‐mediated degradation of aberrant nascent polypeptide (See the working model in Figure S6, Supporting Information).

Listerin directs lysine 48 (K48)‐linked ubiquitin chain to the aberrant nascent polypeptides for proteasome‐mediated destruction dependent on its E3 ubiquitin ligase function in classical RQC,^[^ ^30^ ^]^ however, we found that Listerin decreases TLR4 protein levels by targeting *TLR4* mRNA for cleavaging rather than its protein for post‐translational modification. We observed that Listerin decreases *TLR4* mRNA levels irrespective of its enzymatic activity. However, the RING domain of Listerin is quite essential because the Listerin (ΔR) impedes the *TLR4* mRNA decay. Furthermore, we found that Listerin serves as an indispensable adaptor protein and regulates the re‐localization of IRE1α and the interaction between *TLR4* mRNA and IRE1α, leading to the cleavage of *TLR4* mRNA by IRE1α.

TLR4, a classic pattern recognition receptor, has recently been proven to play a critical role in pro‐inflammatory responses induced by Aβ or Tau in immune cells.^[^ ^31^ ^]^ Upregulated expression of the TLR4 was reported in glial cells surrounding plaques in the postmortem brains of AD patients.^[^ ^32^ ^]^ The inflammation triggered by injection of Aβ in the brain can lead to neuronal death, synaptic loss, and cognitive dysfunction in WT mice, but no such response was observed in TLR4 knockout mice.^[^ ^33^ ^]^ TLR4 activation is connected with the development of AD pathology and cognitive impairment. The reported role of TLR4 in AD is mainly to act as a receptor to recognize and transmit inflammatory signals,^[^ ^31^, ^34^ ^]^ which is modulated by some protein post‐translational modifications (PTMs), such as methylation or ubiquitination in immune responses,^[^ ^35^ ^]^ while little is known about its post‐transcriptional regulation. Yoon. et al. reported that PUM1 interacted with the 3′‐untranslated region of TLR4 and suppressed the translation of *TLR4* mRNA to suppress osteoarthritis.^[^ ^36^ ^]^ Moreover, HNRNPA2B1 mediates the epigenetic regulation of *TLR4* RNA by m^6^A modification, which promotes multiple myeloma proliferation and inhibits apoptosis.^[^ ^37^ ^]^ Our study found that Listerin targets *TLR4* mRNA to regulate brain inflammation in AD. Mechanistically, *TLR4* mRNA is a RIDD substrate of IRE1α, and the cleavage of *TLR4* mRNA mediated by IRE1α dependent on the presence of Listerin based on the in vitro assay. We found that Listerin as a necessary scaffold protein interacts with *TLR4* mRNA and IRE1α. We further identified the cleavage site on *TLR4* mRNA through the prediction and verification of mutation experiments.

Collectively, our study reveals a regulatory role of Listerin that alleviates AD‐related brain inflammation in microglia by impairing the TLR4 signaling pathway. Listerin, as an essential scaffold protein supports IRE1α to cleave *TLR4* mRNA and inhibit the transmission of the TLR4 signaling pathway in microglia. Adenovirus‐mediated overexpression of Listerin in mice attenuated microglia‐mediated inflammatory damage to neuronal cells and thus alleviated AD‐related symptoms. Our study illustrates that Listerin is a promising therapeutic target in AD treatment and may provide an early intervention method that significantly delays neurodegeneration in patients, which further emphasizes the translational potential of targeting this pathway in AD.

## 4 Experimental Section

### Reagents

Antibodies were used as follows: Rabbit anti‐Listerin (1:1000, ab104375, Abcam), Rabbit anti‐His (1:1000, 12 698, Cell Signaling), Rabbit anti‐NF‐κB p65 (1:500, 8242S, Cell Signaling), Rabbit anti‐Phospho‐NF‐κB p65 (1:1000, 3033S, Cell Signaling), Mouse anti‐phospho Tau (Ser202/Thr205) AT8 (1:500, MN1020, Thermo Fisher Scientific), Mouse anti‐NeuN (1:200, MAB377, Millipore), Rabbit anti‐Iba1 (1:1000, ab5076, Abcam), Mouse anti‐Iba1 (1:200, sc‐32725, Santa Cruz), Donkey anti‐Rabbit Alexa Fluor‐647 (1:500, A‐31573, Invitrogen), Goat‐anti‐mouse AlexaFluor‐568 (1:500, A‐11004, Invitrogen), Goat‐anti‐rabbit AlexaFluor‐568 (1:500, A‐11011, Invitrogen), Rabbit anti‐IRE1α (1:1000, 3294, Cell Signaling), Mouse anti‐FLAG (M2) (1:1000, F1804, Sigma), Rabbit anti‐Phospho‐IKKα/β (Ser176/180) (1:1000, 2697, Cell Signaling), Rabbit anti‐IIKKβ (1:1000, ab194519, Abcam), Rabbit anti‐P38 (1:1000, 8690, Cell Signaling), Rabbit anti‐Phospho‐P38 (1:1000, 9215, Cell Signaling), Mouse anti‐TLR4 (1:500, sc‐293072, Santa Cruz), Rabbit anti‐Myc (1:500, A190‐105A, Bethyl), Mouse anti‐Myc (1:1000, TA150121, Origene), Rabbit anti‐Gapdh (1:2000, AB0037, Abways). LPS(L2880), 3‐Methyladenine (M9281) were purchased from Sigma; Bafilomycin A1 (tlrl‐baf1) were purchased from Invivogen; 4µ8C (S7272), STF‐083010 (S7771) were purchased from selleck. Chloroquine (HY‐17589A) and MG132 (HY‐13259) were purchased from MedChemExpress.

### Mice

The *Listerin* ^fl/fl^ mice were obtained from Beijing Biocytogen (Beijing, China) using CRISPR/Cas9 system. Genotyping of mice was completed by PCR using the following primers: forward 5′‐ CGTTGCTTTCGCCTTGGATCAGTTA ‐3′, reverse 5′‐ ATTTAGCTTGTGTGCCTTTGCGTCT ‐3′. Cx3cr1 Cre mice were from Cyagen Biosciences Inc. (Guangzhou, China). The primers for Cx3cr1 Cre transgenic mice genotyping were Cx3cr1‐WF3: 5′‐CCTCAGTGTGACGGAGACAG‐3′, Cx3cr1‐R2: 5′‐GCAGGGAAATCTGATGCAAG‐3′, Cx3cr1‐F1: 5′‐GACATTTGCCTTGCTGGAC‐3′. Listerin^fl/fl^ mice used in the experiments were Cre‐negative littermates of the *Listerin^f^* ^l/fl^ Cx3cr1 Cre mice. All housing, breeding, and procedures were performed in accordance with the general guidelines distributed by the Association for Assessment and Accreditation of Laboratory Animal Care. All the mice were on the C57BL/6 background and were maintained under specific‐pathogen free conditions. The study was performed in accordance with the approval of the Ethics Committee of Scientific Research of Shandong University Qilu Hospital, Jinan, Shandong Province, China (Permit number: KYLL‐2017(KS)‐361).

### Plasmids, siRNA, and Transfection

Human Flag‐Listerin were subcloned into a pCMV2‐Flag vector, human Myc‐IRE1α were subcloned into a pCMV‐N‐Myc vector. His‐TLR2, His‐TLR3, His‐TLR4, and His‐TLR9 were purchased from WZ Biosciences. All mutant plasmids were generated using the KOD‐Plus‐ Mutagenesis Kit (TOYOBO) according to the manufacture's instruction. Small interfering RNAs (siRNAs) against Listerin and IRE1α were designed by Shandong Gene&Bio Co. Ltd. (Shandong, China). Mouse Listerin siRNA: GCAAAUUGCUGCUUAUCAUTT; Human IRE1α siRNA: TCGGGTTTTGGTGTCGTACA. Plasmids were transfected into HEK293T cells cells by Lipofectamine 3000 reagents (Invitrogene). siRNA duplexes were transiently transfected into BV2 cells using Lipofectamine RNAiMAX (Invitrogene).

### Cell Culture

Primary microglia were separated from the newborn mouse brains (P1‐P3) as previously described.^[^ ^38^ ^]^ Briefly, the hippocampi and cortices of newborn mice were mechanically dissected in cold DMEM, then minced and digested with 0.1% Trypsin‐EDTA at 37 °C for 20 min. Trypsin digestion was abolished by adding 10% fetal bovine serum. DNase was used to decompose the sticky DNA. Single cell suspensions were filtered through a 40 µm cell strainer. Cells were pooled in a T‐75 culture flask with Dulbecco's Modified Eagle Medium (DMEM) supplemented with 10% heat inactivated fetal bovine serum (Gibco), 2 mm glutamine (Thermo), 100 U mL^−1^ penicillin, and 100 mg mL^−1^ streptomycin for 12 days of culture. Microglia were obtained by shaking the flask at 100 rpm for 1 h and were counted for plated on non‐treated plates. They were used after another 24 h.

### Hippocampal Neurons and Microglial Co‐Culture

Hippocampal neurons were cultured from E16‐E18 C57BL/6N embryos as described.^[^ ^39^ ^]^ Briefly, the hippocampi were mechanically isolated and digested with 0.05% Trypsin‐EDTA at 37 °C for 15 min. Add 5 µl of DNase and incubate at room temperature for 5 min. Aspirate the medium and wash the tissue twice, add neurobasal medium (Gibco) 2 ml and use 1 ml eppendorf tubes to plating medium to get single cells. Cells were counted and seed into PDL‐coated 24 well plates and maintained in neurobasal medium supplemented with GlutaMAX (Gibco) and B27 (Gibco) for 14 days of differentiation. Microglia and neurons were co‐cultured at a ratio of 1:2 with the transwell system.^[^ ^40^ ^]^ Co‐cultures were treated with recombinant A for 3 days after microglia plating 12 h.

### Immunoblot Analysis

Cells were harvested with cell lysis buffer (Sigma, C2978) containing complete protease inhibitor cocktail (Sigma) and PhosSTOP phosphatase inhibitor (Roche) for 30 min on ice, followed by centrifugation at 12000 x g for 10 min at 4 °C. For the immunoprecipitation assay, cells were lysed with IP buffer (1% (v/v) Nonidet P‐40, 50 mm Tris‐HCl (pH 7.4), 50 mm EDTA, 150 mm NaCl and protease inhibitor mixture). Total protein concentration was quantified using a Pierce BCA Protein Assay Kit according to the manufacturer's instructions (Thermo Scientific, 23 225). The supernatant was incubated with 0.5 µg of the antibodies for 1 h and 35 µl protein A/G plus‐agarose overnight. Cell lysates or immunoprecipitates were incubated at 95 °C for 8 min with 6X SDS loading buffer and separated using SDS–PAGE and analyzed with immunoblotting.

Western blot antibodies were Rabbit anti‐Listerin 1:1000 (Abcam, ab104375), Rabbit anti‐His 1:2000 (Cell Signaling, 12698), Rabbit anti‐NF‐κB p65 1:1000 (Cell Signaling, 8242), Rabbit anti‐Phospho‐NF‐κB p65 1:1000 (Cell Signaling, 3033S), Rabbit IRE1α 1:1000 (Cell Signaling, 3294), Mouse anti‐FLAG (M2) 1:1000 (Sigma, F1804), Rabbit anti‐IIKKβ 1:1000 (Cell Signaling, 8943), Rabbit anti‐Phospho‐IKKβ 1:1000 (Abcam, ab194519), Rabbit anti‐P38 1:1000 (Cell Signaling, 8690), Rabbit anti‐Phospho‐P38 1:1000 (Cell Signaling, 9215), Mouse anti‐TLR4 1:500 (Santa Cruz, SC‐293072), Mouse anti‐Myc 1:1000 (Origene, TA150121), Rabbit anti‐Myc 1:1000 (Bethyl, A190‐105A), Rabbit anti‐Gapdh 1:2000 (Abways, AB0037). Complexes were recovered with magnetic protein A/G beads (Santa Cruz, sc‐2003).

### Quantitative RT‐PCR

Total RNA was extracted using a rapid RNA exaction kit (Aidlab, RN0702) following the manufacturer's instruction manuals, and 1 µg of total RNA was reverse transcribed through the PrimeScript RT reagent Kit (Takara, RR047A). qPCR analysis was accomplished in triplicate wells using the SYBR RT‐PCR kit (Nobelab, Roche, R602) according to the manufacturer's instructions. qPCR primers are listed in Table S1 (Supporting Information).

### ELISA

Brain samples were isolated, weighed and grounded with a pestle to homogenization in PBS containing protease inhibitor (Sigma), then, lysed by ultrasonication and centrifuged at 12000 x g for 10 min at 4 °C. The tissue supernatant was collected and cell culture supernatants were obtained for analysis by ELISA commercial kits according to the manufacturer's instructions.

### CRISPR/Cas9 Knockout

Genome engineering via the CRISPR/Cas9 system involved cloning double‐stranded oligonucleotides that corresponded to the target sequences into the lentiCRISPR V2 vector. The resulting constructs were then co‐transfected into HEK293T cells, which were subsequently selected with puromycin (3 µg mL^−1^) for at least 5 days starting 2 days after transfection. The pool of sorted cells was either directly used in subsequent functional assays or diluted into 96‐well plates by serial dilutions to obtain single clones. The knockout efficiency was examined by RT–qPCR analysis or immunoblotting analysis.

### RNA‐Binding Protein Immunoprecipitation (RIP) Assay

HEK293T cells were transfected with the indicated plasmids for 36 h and washed twice with ice‐cold PBS. RNA‐binding protein immunoprecipitation assays were performed using an RNA Immunoprecipitation Kit (Geneseed, P0102). Cells were lysed in 1 mL Buffer A (1×) with protease inhibitor and RNase inhibitor for 10 min and centrifuged at 14000 x g for 10 min at 4 °C. Then, 100 µl of the supernatant was saved for assay input, and the rest was used for immunoprecipitation according to the manufacturer's instructions. The precipitated RNA and input RNA were detected by RT‐qPCR with appropriate primers to detect the enrichment fold of target RNA.

RIP antibodies were Mouse anti‐Flag antibody (Sigma, F1804) and Mouse anti‐Myc antibody (Bethyl, A190‐105A).

### Protein Purification

Human IRE1α recombinant protein was purified in Baculovirus‐Insect Cells (Sino Biological, 11905‐HNCB). Flag‐Listerin and Flag‐Listerin (ΔR) proteins were purified in HEK293T cells utilizing anti‐FLAG M2 affinity gel (Sigma, A2220) and 3xFlag peptide (Sigma, F4799). Briefly, Flag‐Listerin and Flag‐Listerin (ΔR) constructs were transfected into HEK293T cells by Lipofectamine 3000 reagents (Invitrogen). After 48 h of transfection, cells were collected and lysed in IP buffer (50 mm Tris‐HCl pH 7.4, 50 mm EDTA, 1% NP‐40, 150 mm NaCl, and protease inhibitors) for 30 min. After centrifugation at 12000 x g for 10 min at 4 °C, the obtained supernatant was incubated with 2 µg of Flag agarose beads overnight. The Flag peptide was added to elute the protein for 8 h.

### RNA Cleavage Assay

*XBP1*, *TLR3*, and *TLR4* (WT and Muts) mRNA were transcripted with a standard RNA Synthesis Kit (New England Biolabs, E2050). For the RNA cleavage assay, 1 µg of RNA was cleaved at room temperature by 1 µg of human IRE1α recombinant protein (Sino Biological, 11905‐HNCB) with PBS, Listerin or Listerin (△R) for 30 min in RNA cleavage buffer. For linearization of RNA, the equal volume of formamide was added and then heated at 70 °C for 15 min. The reactants were placed on ice for 10 min and run on 2.5% agarose gel at 4 °C for separation. Gels were imaged on a Bio‐Rad Molecular Imager.

### Fluorescence Microscopy

Fluorescent immunostaining: BV2 cells were silenced for control (siCtrl) or Listerin (siListerin), or microglia were seeded on coverslips in 24‐well plates followed by stimulation with PBS or Aβ (1 µm) for 24 h. The cells were fixed with 4% paraformaldehyde for 20 min, and permeabilized with Triton X‐100 for 20 min. After washing 3 times, the cells were blocked in 2% BSA for 2 h. The cells were labeled with primary antibodies (NF‐κB p65, 1:300, Cell Signaling) overnight at 4 °C. Alexa Fluor 568‐conjugated secondary antibodies (Invitrogen) were incubated for 1 h and dyed with DAPI. After washing and fixing, images were captured with a ZEISS LSM 880 (Zeiss) confocal microscope.

FISH in conjugation with fluorescent immunostaining: FISH was performed following the manufacturer's protocol (LGC Biosearch Technologies). Custom TLR4 probes labeled with Quasar 488 Dye were designed and purchased from Biosearch Technologies. HEK293T cells were transfected with the indicated constructs for 24 h. The fluorescent immunostaining was proceeded with 40 U ml^−1^ RNase inhibitor for primary antibodies (Mouse anti‐Flag, Rabbit anti‐Myc) and secondary antibodies (alexa Fluor 568, alexa Fluor 647). Before the dyeing with DAPI, the cells were incubated with TLR4 probe (12.5 µm) 4 h at 37 °C. After washing and dying with DAPI, images were taken using a ZEISS LSM 880 (Zeiss) confocal microscope.

### Cytotoxicity Assay‐Hoechst/PI Staining

The characteristics and proportion of primary neuron was detected by labeling with Hoechst 33342 and propidium iodide (PI) (Invitrogen). Images were acquired and counted by Zeiss microscope.

### Cytotoxicity Assay‐Lactate Dehydrogenase (LDH)

LDH analysis was finished with an LDH Cytotoxicity Assay Kit (Beyotime). The absorbance was analyzed at 490 nm. The LDH release activity was analyzed as follows:

(1) $$\left[\mathrm{ODsample}-\mathrm{ODblank}\right]/\left[\mathrm{ODmax}-\mathrm{ODblank}\right].$$

### Cytotoxicity Assay‐TUNEL

The DNA fragmentation of neurons was detected by a TUNEL cell apoptosis detection kit (Beyotime). The treated cells were fixed with 4% paraform phosphate buffer saline, washed with PBS, then permeabilized by 0.2% Triton X‐100 and stained according to the manufacturer's instructions.

### Stereotaxic Surgery and Injection

Mice were deeply anesthetized with isoflurane and immobilized in a stereotaxic frame. Aβ_1‐42_ protein was purchased from Sigma‐Aldrich and oligomers was prepared as described previously.^[^ ^18a^ ^]^ Briefly, freeze‐drying Aβ_1‐42_ peptides were dissolved in hexafluoroisopropanol (HFIP) and incubated overnight at room temperature to obtain monomers. Evaporate HFIP with nitrogen to form a film, and Aβ was dissolved again in DMSO. Aβ_1‐42_ monomer solution (50 µm) was stored at ‐20 °C as a reserve solution. Aβ_1‐42_ oligomers (50 µm) from Aβ1‐42 monomer solution was incubated in darkness at 37 °C for 48 h to obtain. Mice were intracranially injected with PBS, Aβ_1‐42_ oligomers (1 µg per mouse), LPS (5 µg per mouse), or adenovirus (Adv‐GFP or Adv‐Listerin‐GFP, GeneChem) into the bilateral entorhinal cortices according to the predetermined procedure. Mice were anesthetized with isoflurane (2%) and injections were performed at the following coordinates (anterior/posterior (A/P): −4.7 mm; medial/lateral (M/L): ±3.3 mm; dorsal/ventral (D/V): −2.0 mm from brain surface). All mice were injected at a rate of 0.2 µl min^−1^ by the glass micropipette. The skin at the injection site was sutured, and mice were maintained on a warm pad for recovery.

### Animal Motor Assessment—Y‐Maze Test

Spatial memory during exploratory behavior was tested in a Y‐shaped maze as previously described.^[^ ^41^ ^]^ The Y‐shaped maze was made of a gray wood material consisting of three identical detection arms with equal angles (63006, RWD Life Science Co., Ltd., China), which are 50 centimeters tall. The video tracking system (Smart 3.0, Panlab, Spain) was used to track the mice. After the mice became accustomed to the operating room at least 1 h before testing, they were placed at the end of one arm and allowed to explore the maze freely through the maze during an 8 min session. After each test, the apparatus was cleaned with 75% ethanol to remove olfactory cues. The percentage of spontaneous changes, expressed as change scores, was calculated as follows:

(2) $$\left(\mathrm{Alternations}/\mathrm{Total}\;\mathrm{Arm}\;\mathrm{Entries}\right)-2\times 100=\mathrm{percent}\;\mathrm{alternation}.$$

### Animal Motor Assessment—Open Field Test

To investigate differences in generalized locomotor activity, mice were subjected to the open field test as previously described.^[^ ^42^ ^]^ Before the behavioral test, each mouse was brought into the room and acclimatized for at least 1 h. Mice were placed in the center of a custom‐built 40 × 40 cm arena (63008, RWD Life Science Co., Ltd., China). All the behaviors of each mouse were recorded and analyzed using a Smart Video Tracking System (Smart 3.0, Panlab, Spain) for 10 min. After each test, the apparatus was cleaned with 75% ethanol to remove olfactory cues. The mouse total movement distance and the percentage of total time spent in the central area were analyzed.

### Novel Object Recognition Test (NOR)

Novel object recognition test was performed as previously described.^[^ ^43^ ^]^ Mice were familiarized with the behavioral chamber for 1 h prior to detection to minimize experimental error. First, the mice were subjected to the habituation period (5 min), in which were placed in a behavioral detection box (40 × 40 × 40 cm) without any objects available for free exploration for 5 min. Then, the mice were subjected to a 10 min training period. Two same objects were provided at equidistance left and right of the box. Mice were allowed in the box and learned the objects for 10 min. Last, mice were subjected to a 10 min test period. One object was replaced with another totally different object and placed in the original location. The mice were t allowed to explore freely for 10 min. After each test, the box was wiped with 75% ethanol to remove leftover odors from the previous experiment. Memory was evaluated as follows:

(3) $$\begin{array}{ccc} & & \mathrm{discrimination}\;\mathrm{index}\;\%=\mathrm{exploration}\;\mathrm{time}\;\mathrm{for}\;\mathrm{new}\;\mathrm{objects}\;(S)/\\ & & \;\times \;(\mathrm{exploration}\;\mathrm{time}\;\mathrm{for}\;\mathrm{new}\;\mathrm{objects}\;(s)+\mathrm{old}\;\mathrm{object}\;(S))\times 100\%. \end{array}$$

### Fluorescence Immunohistochemistry (IHC)

Cardiopulmonary perfusion of mice was performed under pentobarbital anesthesia (50 mg kg^−1^, i.v.) with normal saline followed by 4% paraformaldehyde in 0.1 M phosphate buffer. Brains were fixed overnight at 4 °C in the same fixative solution. For fluorescence IHC, brains were cryoprotected by placing them in 30% sucrose before freezing with dry ice powder. Coronal sections (20 µm) were cut and mounted on glass slides. Slides were then heated in 10 mm citrate buffer, pH 6.0, at 97 °C for 15 min, followed by treatment with 1% Triton X‐100, 2× Block Ace solution, and 10% NDS for 1 h. Sections were then incubated with mouse anti‐AT8 (Thermo Fisher Scientific, MN1020), anti‐Iba1 (Abcam, ab5076), or rabbit anti‐NeuN (Millipore, MAB377) overnight at room temperature. After hatching, slides were incubated with Goat‐anti‐mouse AlexaFluor‐568 or Goat‐anti‐ rabbit AlexaFluor‐568 (Invitrogen) for 2 h at room temperature, slides were dyed with DAPI (Life Technologies). Slides were imaged with a VS120 microscope (Olympus).

### Quantification and Statistical Analysis

Quantification was described in the method details and figure legends. Statistical analysis was completed using GraphPad Prism 8.0 and statistical significance is exhibited in the relevant figures and figure legends. The number of samples was shown and described in the figure legends. Error bars represent the standard error of the standard deviation (SD) as indicated in the figure legend, and the statistical tests were defined in the figure legends. Experimentalists remained blinded to experimental conditions whenever possible during data acquisition or quantification.

## Conflict of Interest

The authors declare no conflict of interest.

## Author Contributions

C.G. and B.L. conceived and designed the study. F.Q. performed most of the experiments with help from R.C., X.B., and J.Y.; W.S., Y.Z., X.Q., and W.Z. contributed to the discussion and provided reagents. C.G. and B.L. supervised the study. C.G., B.L., and F.Q. analyzed the data. C.G., B.L., and F.Q. wrote the paper.

## Supporting information



Supporting Information

## Data Availability

The data that support the findings of this study are available in the supplementary material of this article.
