# Supplementary material for: Listerin Alleviates Alzheimer's Disease through IRE1‐mediated Decay of TLR4 mRNA
Source: Adv Sci (Weinh). 2025 May 31;12(32):e14956. doi: 10.1002/advs.202414956 (PMC12407295; doi:10.1002/advs.202414956)
Supplement: Supplementary file 1 — Supporting Information [file ADVS-12-e14956-s001.docx]

**Supplemental information**

**Listerin****alleviates** **Alzheimer's disease through IRE1-mediated decay of *TLR4* mRNA**

Fei Qin, Runyu Cao, Xuemei Bai, Jiahua Yuan, Wanwei Sun, Yi Zheng, Xiaopeng Qi, Wei Zhao, Bingyu Liu, Chengjiang Gao

*** Corresponding authors:**

**E-mail: cgao@sdu.edu.cn and liubingyu@sdu.edu.cn**

**Supplemental table S1. Primers for qRT-PCR**

| **Gene** | **Forward** | **Reverse** |
| --- | --- | --- |
| Mouse *TNFα* | GCCACCACGCTCTTCTGTCT | TGAGGGTCTGGGCCATAGAAC |
| Mouse *IL*6 | ACAACCACGGCCTTCCCTAC | CATTTCCACGATTTCCCAGA |
| Mouse *IL12b* | ATGTGGAATGGCGTCTC | GTCTCCTCGGCAGTTGG |
| Mouse *IL1β* | ACCTTCCAGGATGAGGACATGA | AACGTCACACACCAGCAGGTTA |
| Human *TNFα* | TGAAGAGGACCTGGGAGTAGAT | TGAAGAGGACCTGGGAGTAGAT |
| Human *IL*6 | TGCAATAACCACCCCTGACC | TGCGCAGAATGAGATGAGTTG |
| Human *IL12b* | CACAAAGGAGGCGAGGTTCT | TTTGGGTTCTTTCTGGTCCTT |
| Human *IL1β* | TGATGGCTTATTACAGTGGCA | GGTCGGAGATTCGTAGCTGG |
| Mouse *TLR4* | GTTCCTTGACCCACT | TATTTGACACCCTCC |
| Human *TLR4* | TGGTGTCCCAGCACTTCATC | CTGTCCTCCCACTCCAGGTA |
| Human *TLR2* | TTG TGC CCA TTG CTC TTT TCA C | TGG CAT TGT CCA GTG CTT CA |
| Human *TLR3* | GCAAAAGATTCAAGGTACATCATGC | CCTCTTCGCAAACAGAGTGC |
| Human *TLR9* | TCCTGCCCAAACTGGAAGTC | TAAGGTTGAGCTCTCGCAGC |


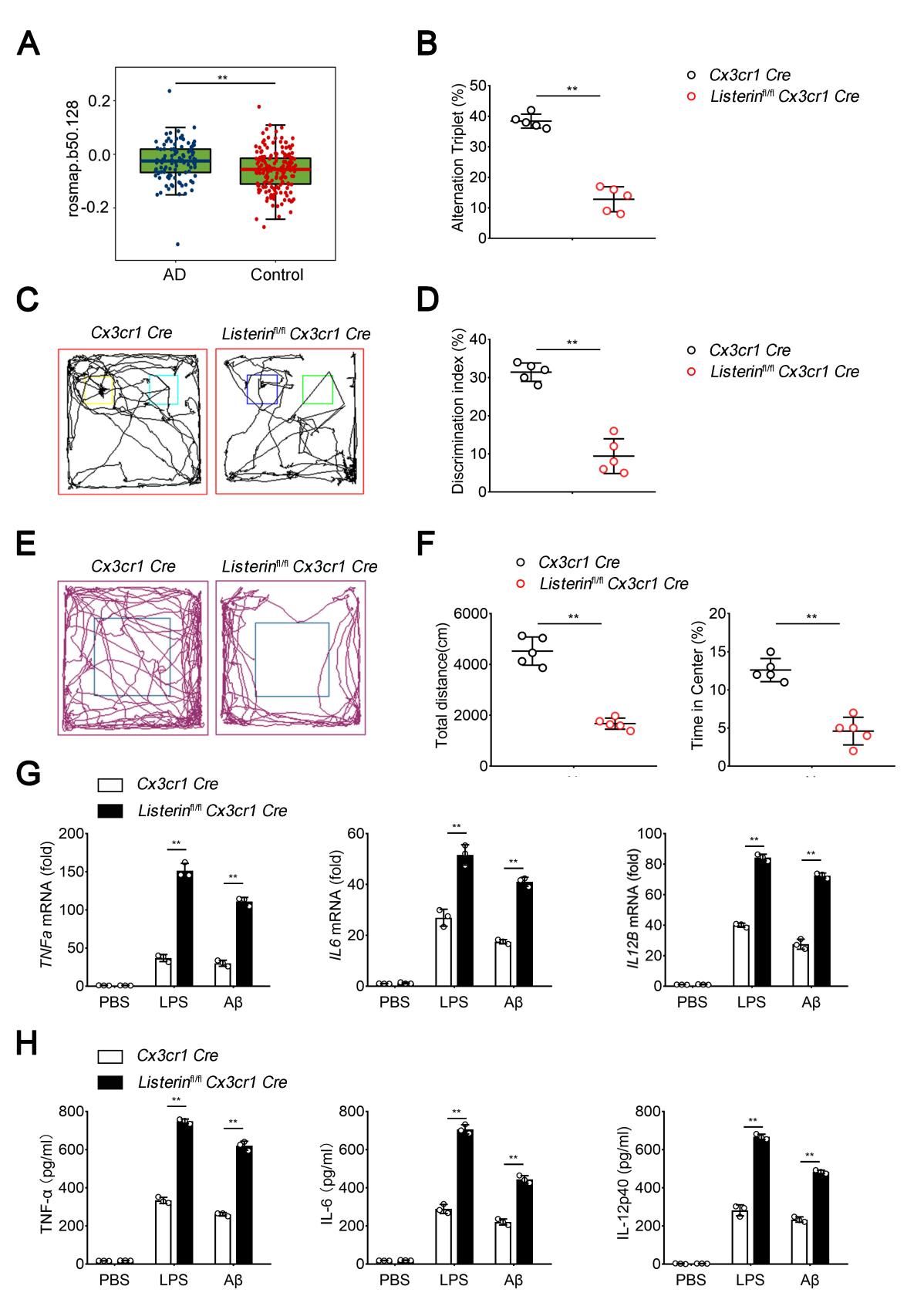
**Figure S1. The mean expression of Listerin in the AD group and the control group**

**A.** Listerin eigenprotein levels by case status. Data from the previous study by TMT-MS-based quantitative proteomics. **B**-**F.** 12 days after Aβ injection in *Cx3cr1 Cre* and *Listerin*^fl/fl^ *Cx3cr1 Cre* mice, Y maze (b), Novel object recognition (c, d) or Open field test (e, f) was performed. **G.** Primary microglia prepared from *Cx3cr1 Cre* and *Listerin*^fl/fl^ *Cx3cr1 Cre* mice were stimulated with LPS, or Aβ for 24 h, cell lysates were collected and *Tnfa*, *Il6* and *IL12b* mRNA were quantified by qPCR. **H.** Supernatant in (g) were collected and TNF-α, IL-6 and IL-12p40 were quantified by ELISA. Statistical analysis was analyzed by unpaired student's t-test. Data are presented as means ± SD. ***P* < 0.01.


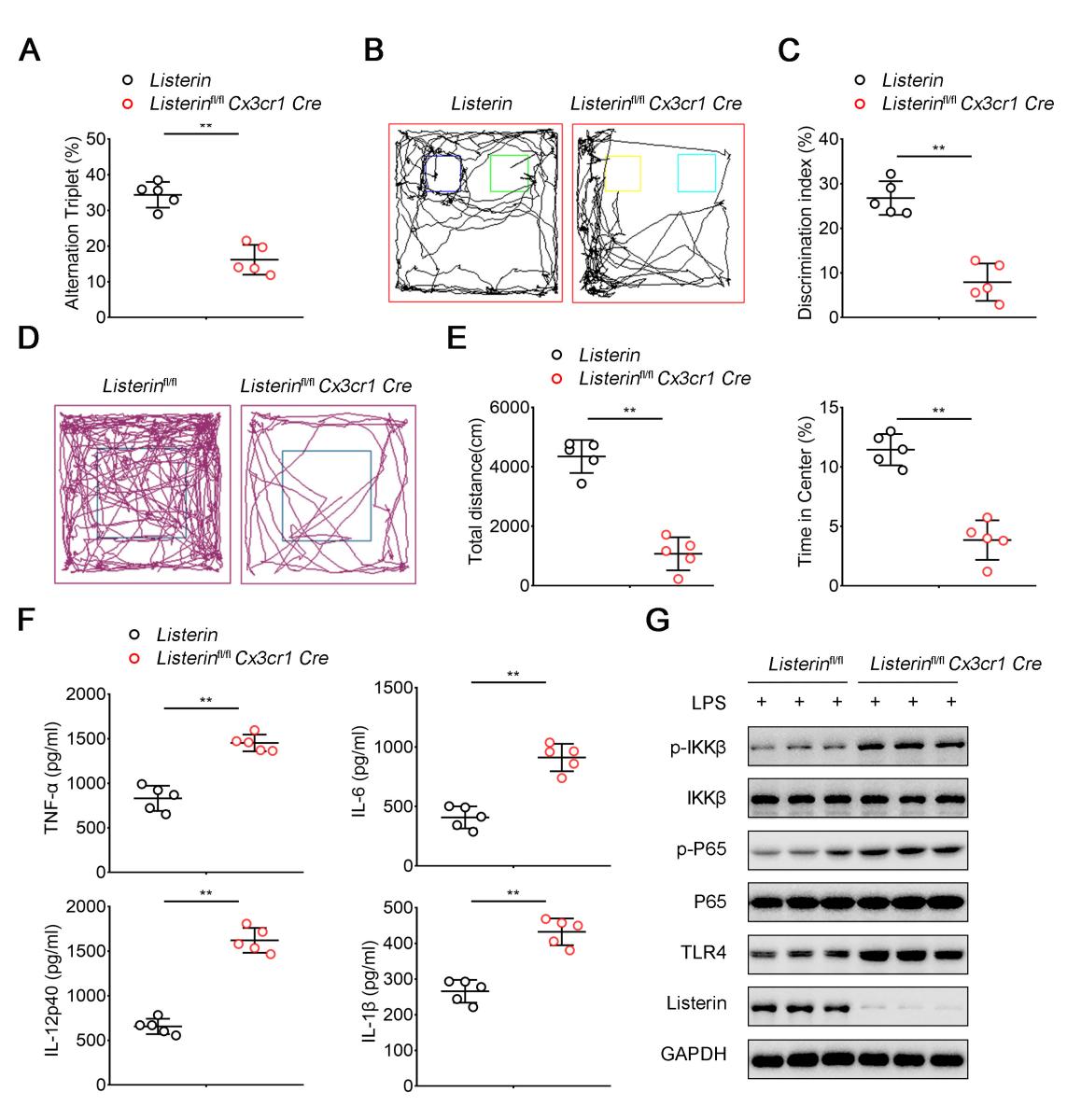
 **Figure S2. *Listerin* deletion in microglia exacerbates LPS induced disease progression in *vivo.* A-E.** 12 days after LPS injection in *Listerin*^fl/fl^ and *Listerin*^fl/fl^ *Cx3cr1 Cre* mice, Y maze (a), Novel object recognition (b and c) or Open field test (d, e) was performed. **F.** TNF-α, IL-6, IL-12p40 and IL-1β concentrations in the hippocampi of *Listerin*^fl/fl^ and *Listerin*^fl/fl^ *Cx3cr1 Cre* mice after LPS injection measured by ELISA. **G.** Representative immunoblots of phospho-IKKβ and phospho-P65 in the hippocampi of *Listerin*^fl/fl^ and *Listerin*^fl/fl^ *Cx3cr1 Cre* mice after LPS injection. Statistical analysis was analyzed by unpaired student's t-test. Data are presented as means ± SD. ***P* < 0.01.

**
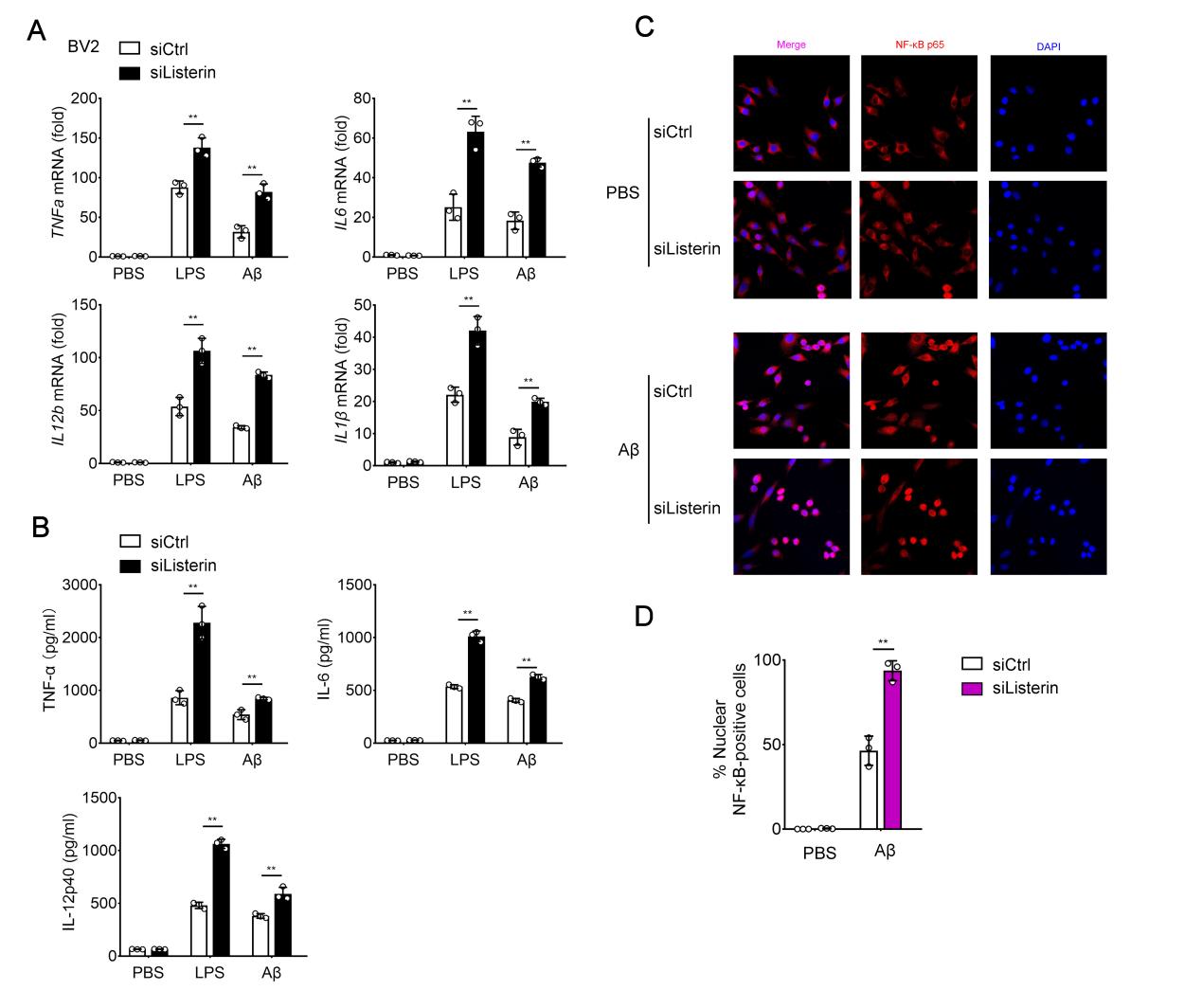
Figure S3. Listerin inhibits AD-related neuroinflammation in BV2 cells. A.** BV2 cells were silenced for control (siCtrl) or Listerin (siListerin) for 48 h followed by stimulated with LPS or Aβ for 24 h, cell lysates were collected and *TNFa*, *IL6* *IL12b* and *IL1β* mRNA were quantified by qPCR. **B.** Supernatant in (a) were collected and TNF-α, IL-6 and IL-12p40 were quantified by ELISA. **C.** Confocal images of nuclear translocation of NF-κB p65 (red) after Aβ treatment in BV2 cells silenced for control (siCtrl) or Listerin (siListerin) for 48 h followed by stimulated with Aβ for 24 h. Scale bars, 100 μm. **D.** Quantitative analyses of % nuclear NF-κB-positive microglia in (c). Statistical analysis was analyzed by unpaired student's t-test. Data are presented as means ± SD. ***P* < 0.01.


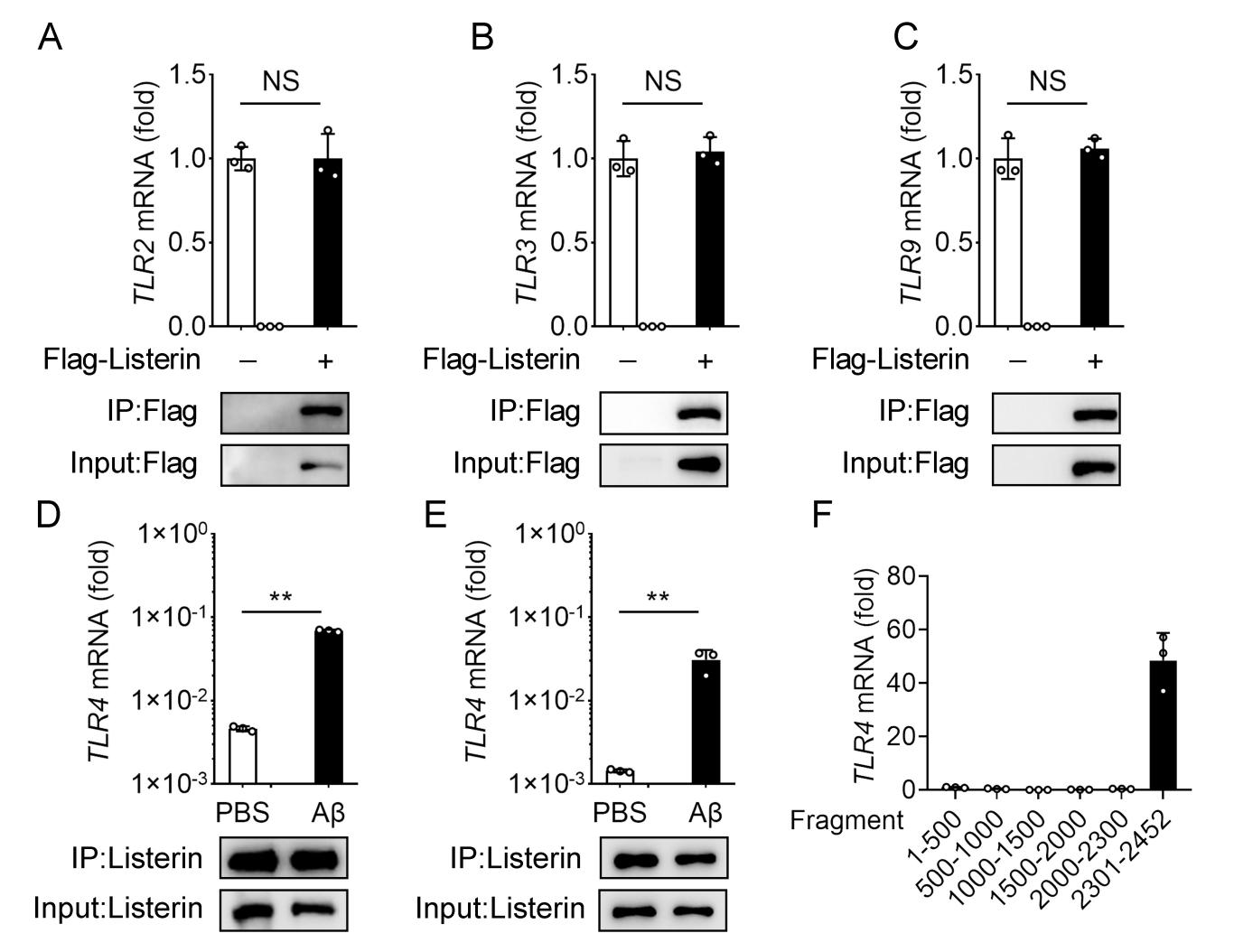
 **Figure S4. Listerin does not interact with *TLR2*, *TLR3* or *TLR9* mRNA. A-C**. RIP assay for *TLR2* (a), *TLR3* (b) or *TLR9* (c) mRNA in HEK293T cells transfected with His-TLR2 (a), His-TLR3 (b) or His-TLR9 (c) together with Vector or Flag-Listerin constructs. Normalized data were shown as relative fold enrichment to the control group. (D) RIP assay for *TLR4* mRNA in BV2 cells with or without Aβ stimulation. (E) RIP assay for *TLR4* mRNA in primary microglia cells with or without Aβ stimulation. (F) RIP assay for fragments of *TLR4* mRNA with Listerin. Statistical analysis was analyzed by unpaired student's t-test. Data are presented as means ± SD. NS, no significance. ***P* < 0.01.

**Figure S5. IRE1α is required for Listerin mediated TLR4 degradation. A.** Representative immunoblots of HEK293T cells transfected with His-TLR4 together with vector or Flag-Listerin constructs for 20 h and then treated with 4μ8C (10 μM) or STF-083010 (10 μM) for
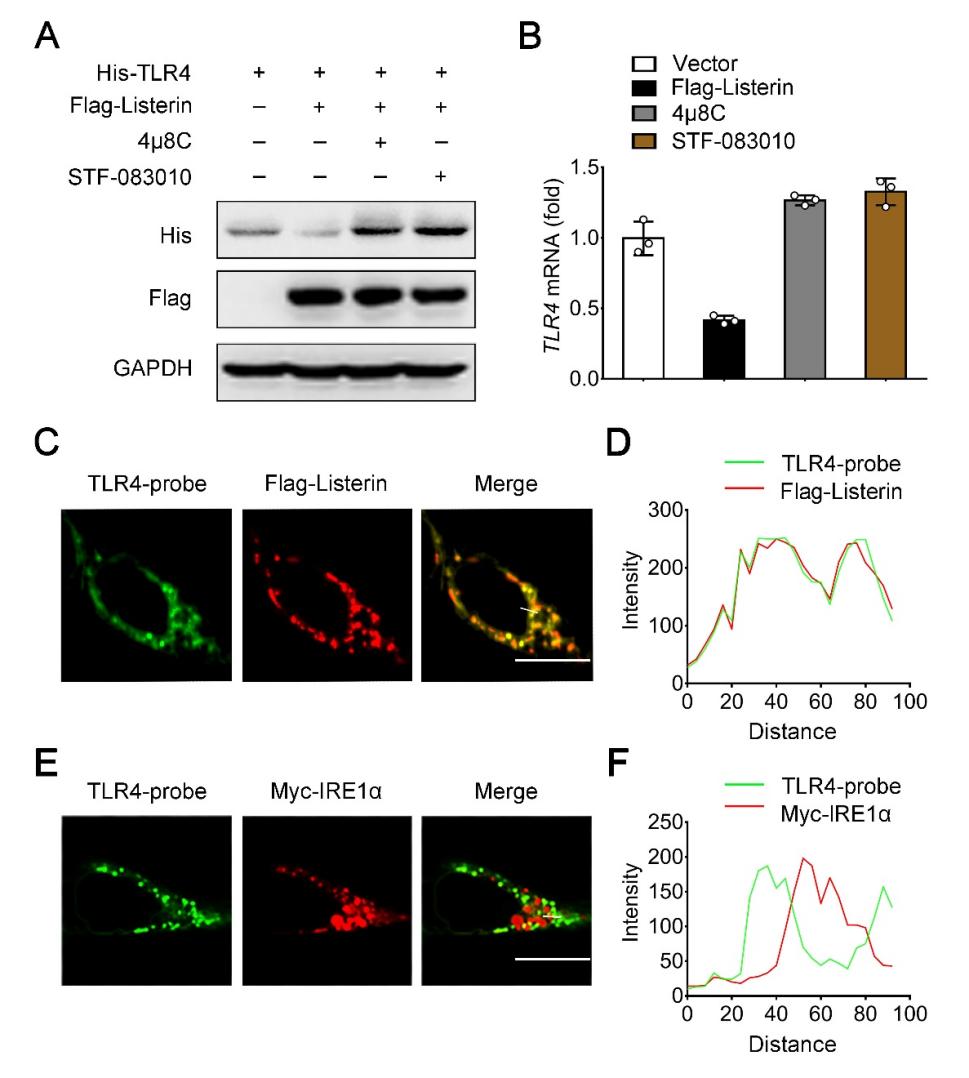
10 h. **B.** Cells treated as in (a) were collected and *TLR4* mRNA were quantified by qPCR. **C**, **D.** Representative images (c) and intensity profiles (d) of FISH for *TLR4* mRNA and fluorescence immunostaining for Flag-Listerin in HEK293T cells transfected with His-TLR4 together with Flag-Listerin constructs. **E, F.** Representative images (e) and intensity profiles (f) of FISH for *TLR4* mRNA and fluorescence immunostaining for Myc-IRE1α in HEK293T cells transfected with His-TLR4 together with Myc-IRE1α constructs. Scale bars, 20 μm.


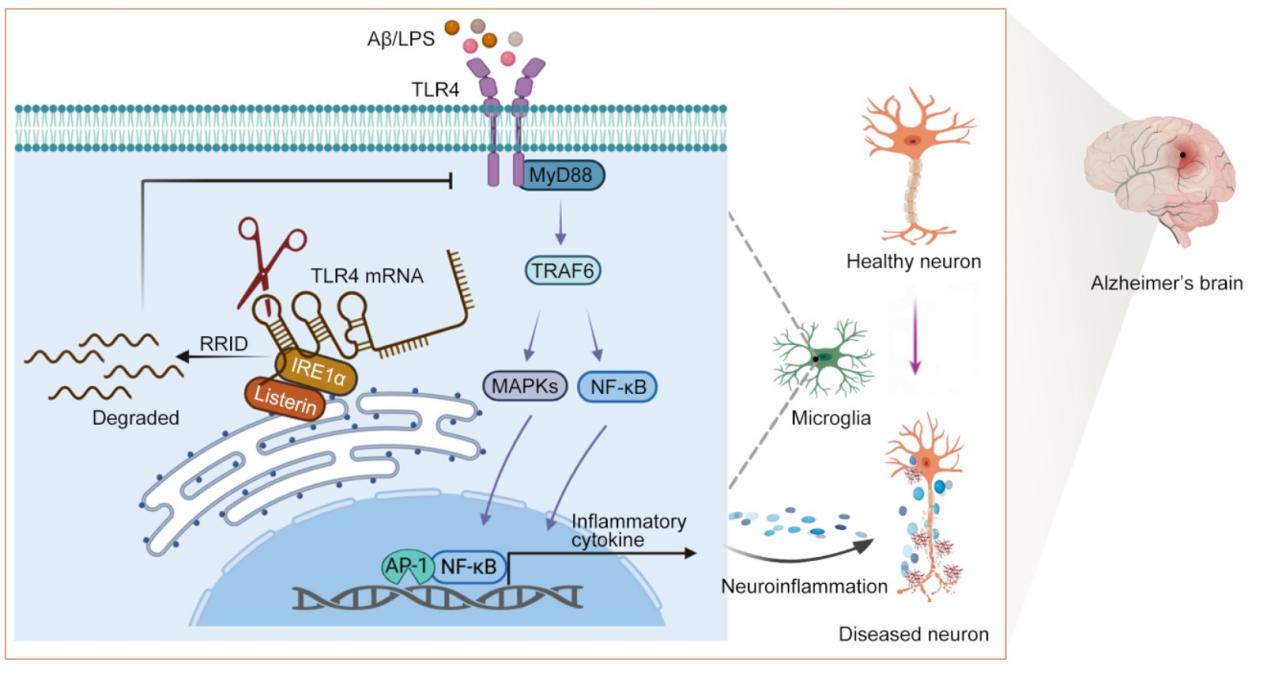
**Figure S6.** The working model of Listerin alleviates Alzheimer's disease through IRE1-mediated decay of *TLR4* mRNA.
